# 60 YEARS OF NEUROENDOCRINOLOGY: The hypothalamo-prolactin axis

**DOI:** 10.1530/JOE-15-0213

**Published:** 2015-08

**Authors:** David R Grattan

**Affiliations:** 1 Centre for Neuroendocrinology and Department of Anatomy, University of Otago, PO Box 913, Dunedin 9054, New Zealand; 2 Maurice Wilkins Centre for Molecular Biodiscovery, Auckland, New Zealand

**Keywords:** prolactin, tuberoinfundibular dopamine neurons, pregnancy, lactation, prolactin-releasing factor

## Abstract

The hypothalamic control of prolactin secretion is different from other anterior pituitary hormones, in that it is predominantly inhibitory, by means of dopamine from the tuberoinfundibular dopamine neurons. In addition, prolactin does not have an endocrine target tissue, and therefore lacks the classical feedback pathway to regulate its secretion. Instead, it is regulated by short loop feedback, whereby prolactin itself acts in the brain to stimulate production of dopamine and thereby inhibit its own secretion. Finally, despite its relatively simple name, prolactin has a broad range of functions in the body, in addition to its defining role in promoting lactation. As such, the hypothalamo-prolactin axis has many characteristics that are quite distinct from other hypothalamo-pituitary systems. This review will provide a brief overview of our current understanding of the neuroendocrine control of prolactin secretion, in particular focusing on the plasticity evident in this system, which keeps prolactin secretion at low levels most of the time, but enables extended periods of hyperprolactinemia when necessary for lactation. Key prolactin functions beyond milk production will be discussed, particularly focusing on the role of prolactin in inducing adaptive responses in multiple different systems to facilitate lactation, and the consequences if prolactin action is impaired. A feature of this pleiotropic activity is that functions that may be adaptive in the lactating state might be maladaptive if prolactin levels are elevated inappropriately. Overall, my goal is to give a flavour of both the history and current state of the field of prolactin neuroendocrinology, and identify some exciting new areas of research development.

## Introduction

When Geoffrey Harris wrote his influential monograph on ‘Neural Control of the Pituitary Gland’, it was already apparent that prolactin, or ‘lactogenic hormone’ as he referred to it, might be controlled differently to the other adenohypophyseal hormones. Harris convincingly built the case that humeral factors from the hypothalamus control secretion of anterior pituitary hormones, correctly predicting the existence of hypothalamic ‘releasing factors’ that mediate the neural control of pituitary secretions (Harris 1948). Despite the clear evidence that neural stimuli (such as suckling) could stimulate prolactin secretion, however, Harris noted that cutting the pituitary stalk did not abolish lactation (Dempsey & Uotila 1940), leading to the inevitable conclusion that a hypothalamic ‘prolactin-releasing factor’ was not necessary to stimulate prolactin secretion. Indeed, it was subsequently shown in hypophysectomised rats that ectopic pituitary grafts were able to maintain corpus luteum function (Everett 1954) and lactation (Cowie *et al*. 1960), demonstrating hypothalamic-independent secretion of prolactin from the grafts. At first impression, this appeared to contradict Harris' principle of neural control of the anterior pituitary. But further observations identified the fact that hypothalamic regulation was indeed critical for normal prolactin secretion, as Harris predicted, but that the mode of control was different. Everett found that while pituitaries located away from the hypothalamus would induce pseudopregnancy, a marker of elevated prolactin secretion in rodents, normal cycles (and therefore normal low levels of prolactin) would resume if transplanted pituitaries were re-vascularised by portal vessels under the median eminence (Nikitovitch-Winer & Everett 1958). Two groups independently demonstrated that extracts of the median eminence/infundibular region could inhibit prolactin secretion from anterior pituitary cells *in vitro* (Pasteels 1963, Talwalker *et al*. 1963). Subsequently, it was shown that median eminence lesions (Arimura *et al*. 1972) or destruction of the pituitary stalk (Kanematsu & Sawyer 1973) resulted in elevated prolactin secretion (accounting for the pseudopregnancies described earlier by Everett). Thus, it was proven that the hypothalamus was essential for the regulation of prolactin secretion, but that it primarily exerted an inhibitory influence.

This brief review will summarise our current understanding of the hypothalamic control of prolactin secretion, and the neuroendocrine functions of prolactin, highlighting (admittedly selected) areas of current research interest. From the somewhat contrary beginnings previously introduced, the neural control of prolactin secretion, and indeed the whole hypothalamo-prolactin axis, continues to prove itself a bit different from other hypothalamo-pituitary systems. Not only is the hypothalamic regulation predominantly inhibitory, as opposed to stimulatory, it also involves a catecholamine neurotransmitter, dopamine, rather than the more typical peptide hypothalamic hormones involved in regulating all other pituitary systems. Prolactin is also the only anterior pituitary hormone that does not have an endocrine target tissue, and therefore lacks a classical hormonal feedback system. It is regulated, instead, by a short loop feedback whereby prolactin itself stimulates the secretion of the inhibitory factor, dopamine. Finally, despite its rather simple and one-dimensional name, prolactin does much more than simply PROmote LACTation. It is now recognised as a pleiotrophic hormone with arguably the widest range of physiological actions of any extracellular signalling molecule in the body.

## Neuroendocrine control of prolactin secretion

### Dopamine as a prolactin-inhibitory hormone

**Figure 1:**
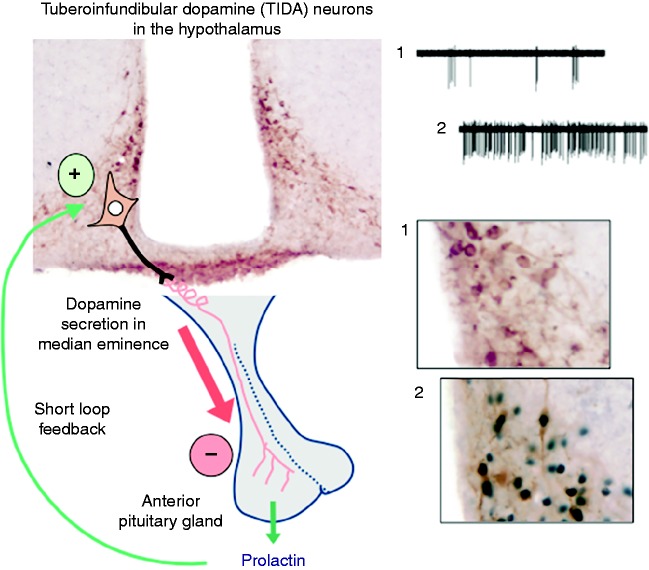
Diagrammatic representation of the neuroendocrine regulation of prolactin secretion. Anterior pituitary prolactin release is inhibited by dopamine coming from the tuberoinfundibular dopamine neurons (shown in the coronal section on the top left using immunohistochemistry against tyrosine hydroxylase, brown) whose cell bodies are found in the arcuate nucleus of the hypothalamus, with axons projecting to the external layer of the median eminence. Images on the right show examples of both rapid feedback (electrophysiological activation) and delayed feedback (phosphorylation of STAT5, black nuclear staining) in TIDA neurons. In each example, 1) illustrates prior to prolactin treatment, and 2) after administration of prolactin (reproduced, with permission, from Brown RS, Piet R, Herbison AE & Grattan DR (2012) Differential actions of prolactin on electrical activity and intracellular signal transduction in hypothalamic neurons. *Endocrinology* **153** 2375–2384. Copyright 2012 The Endocrine Society). Prolactin stimulates dopamine secretion, to inhibit its own secretion by short loop feedback.

Even after the clear demonstration of inhibitory regulation of prolactin secretion in the 1950s, the search for the inhibitory hormone mediating this action was controversial. All hypothalamic hormones identified to date had been peptides, and the expectation was that ‘prolactin-inhibitory factor’ would also be a peptide. Initial clues that this might not be the case came from observations that drugs such as reserpine, which depletes endogenous catecholamines, induced pseudopregnancy in rats (Barraclough & Sawyer 1959), indicative of elevated prolactin. It was assumed, however, that the functional role of catecholamines were as neurotransmitters acting in the hypothalamus to regulate the release of a hypothalamic hormone (Kanematsu *et al*. 1963). Based on the evidence of dopaminergic nerve terminals in the median eminence (Fuxe 1963), McLeod proposed that dopamine may be released into the pituitary portal system, and thereby acting as a hypothalamic hormone (as distinct from its neurotransmitter role in other systems) (MacLeod *et al*. 1970). He demonstrated that dopaminergic agonists were effective at suppressing prolactin secretion *in vivo*, and perhaps more importantly, that dopamine could inhibit prolactin secretion from isolated pituitary glands (MacLeod *et al*. 1970). Dopamine was subsequently detected in the pituitary portal blood (Kamberi *et al*. 1970), and Porter's group (and others) found that variations of levels of dopamine in the portal blood accounted for changes in prolactin secretion in various physiological conditions (Ben-Jonathan *et al*. 1977, 1980, Gibbs & Neill 1978, De Greef & Neill 1979). Dopamine receptors were identified on lactotroph cells in the anterior pituitary (Mansour *et al*. 1990). The observation that mice lacking the dopamine D2 receptor are hyperprolactinemic (Kelly *et al*. 1997, Saiardi *et al*. 1997), clearly demonstrated the critical role of dopamine in suppressing endogenous prolactin secretion (see Fig. 1).

The dopamine neurons that control prolactin secretion are located within the arcuate nucleus of the hypothalamus. While it seems likely that they serve functionally as a single population, these neurons have been subdivided into three sub-populations based on the anatomy of their projections: the tuberoinfundibular (TIDA), tuberohypophyseal (THDA), and periventricular hypophyseal (PHDA) dopaminergic neurons (Freeman *et al*. 2000). TIDA neurons arise from the dorsomedial arcuate nucleus and project to the external zone of the median eminence (Bjorklund *et al*. 1973). The other two populations have their cell bodies located slightly more rostrally, but their projections pass in the hypothalamo-hypophyseal tract through the median eminence to the hypophysis. The THDA neurons originate in the rostral arcuate nucleus and project to the intermediate and neural lobes of the pituitary gland (Fuxe 1964, Holzbauer & Racke 1985), while the PHDA neurons originate in the periventricular nucleus, with axons terminating in the intermediate lobe (Goudreau *et al*. 1992). The TIDA neurons produce the classical hypothalamic hormone secretion into the pituitary portal blood vessels, while THDA and PHDA neurons contribute to basal regulation of prolactin secretion, after transport of dopamine to the anterior pituitary gland through short portal vessels from the neurohypophysis (Peters *et al*. 1981). While anatomically distinct, there is considerable overlap in their dendritic fields (van den Pol *et al*. 1984), and all three populations appear to be regulated similarly. For example, all are stimulated by prolactin (DeMaria *et al*. 1999). Hence, it is reasonable to consider them as a functional unit of prolactin-inhibiting neurons.

Electrophysiological studies of hypothalamic dopamine neurons in the rat have described TIDA neurons as exhibiting a robust oscillation between hyperpolarized and depolarized states, with periodicity of about 20 s, and a spontaneous firing rate of ∼4 Hz during the depolarized ‘up-state’. Remarkably, the TIDA neurons were found to show a synchronous pattern of firing suggestive of an interconnected network, dependent on functional expression of gap junctions (Lyons *et al*. 2010). Taking advantage of transgenic technologies to label dopaminergic neurons with fluorescent tags, studies of TIDA electrical activity have also been completed in brain slices from mice (Brown *et al*. 2012, Romano *et al*. 2013), showing a similar pattern of firing to that seen in the rat, although only a small proportion of TIDA neurons showed the phasic oscillations in this model. Importantly, one of these latter studies demonstrated that patterns of firing of an individual TIDA neuron were reflected in the pattern of dopamine release from the population, as measured using *in vivo* amperometry in the median eminence (Romano *et al*. 2013). These data support the concept postulated by Lyons *et al*. (2010), that the neurons act as a synchronous network to release dopamine in a pulsatile or phasic fashion.

Of the five dopamine receptors, the two members of the D2-like receptor family, D2 and D4 are found in the pituitary gland (Valerio *et al*. 1994, Matsumoto *et al*. 1995) and it is through these D2-like receptors that dopamine acts to inhibit lactotroph cell function (Mansour *et al*. 1990, Ben-Jonathan & Hnasko 2001). Uniquely among anterior pituitary cells, lactotrophs display spontaneous electrical activity in the absence of hypothalamic stimulation and Ca^2+^ influx through voltage-gated Ca^2+^ channels (VGCC) stimulates to prolactin secretion (Gregerson 2006). This accounts for the high levels of basal prolactin secretion, and is consistent with a regulatory mechanism primarily based on inhibition. Dopamine inhibits calcium influx resulting in membrane hyperpolarisation (Gregerson *et al*. 1994, Gregerson 2003) and reduced prolactin secretion (Lledo *et al*. 1990). In addition to its effect on secretion, dopamine-induced suppression of adenylate cyclase leads to a reduction in prolactin gene expression (Maurer 1982, Elsholtz *et al*. 1991, Ishida *et al*. 2007). Dopamine also has a significant role to regulate lactotroph proliferation, as demonstrated in cultures of pituitary cells (Ishida *et al*. 2007), as well as *in vivo* by suppression of oestradiol-induced proliferation (Borgundvaag *et al*. 1992). When dopamine levels are increased, such as caused by the loss of the dopamine transporter, there is a severe post-natal reduction in lactotroph proliferation leading to a dramatic reduction in the number of lactotrophs by 8 weeks of age (Bosse *et al*. 1997). In contrast, there is marked lactotroph hyperplaisia following loss of the D2 receptor (Kelly *et al*. 1997, Saiardi *et al*. 1997), leading to the formation of prolactinomas. This is exacerbated by age, and more prevalent in females than males (Saiardi *et al*. 1997, Asa *et al*. 1999). A bias towards lactotroph hyperplasia and more rapid generation of pituitary tumours in females may be expected from the direct actions of estradiol to stimulate prolactin production by lactotrophs, but this may not be the sole factor leading to the increased female hyperplasia. Gonadectomy has been shown to reduce lactotroph hyperplasia and tumour formation in D2 knockout mice, but that this could not be fully rescued by estradiol replacement, suggesting that ovarian factors other than estradiol may contribute to the proliferation of lactotrophs (Hentges & Low 2002).

### Prolactin regulation of dopamine neurons: short loop feedback

As previously mentioned, the hypothalamo-prolactin system does not have a specific endocrine target, and therefore lacks the classical hormone-mediate negative feedback pathway described for all other anterior pituitary hormones. Nevertheless, it is still regulated in a negative feedback manner, with prolactin itself providing the afferent signal in a process known as short-loop feedback. The presence of prolactin receptors on dopamine neurons (Lerant & Freeman 1998, Grattan 2001, Kokay & Grattan 2005) was predicted by early neurochemical studies that showed that exogenous prolactin stimulated hypothalamic dopamine synthesis (Hokfelt & Fuxe 1972) and turnover (Eikenburg *et al*. 1977, Annunziato & Moore 1978), increased dopamine metabolism in the median eminence (Lookingland *et al*. 1987) and promoted dopamine secretion into the pituitary portal blood (Gudelsky & Porter 1979). In contrast, hypoprolactinemia induced by administration of dopamine agonists resulted in suppression of dopamine secretion (Arbogast & Voogt 1991*a*), indicating that the basal activity of these neurons is dependent on the endogenous levels of prolactin present in the blood. Using these biochemical indices of activity of TIDA neurons, the time course of prolactin action was described as having a ‘rapid’ component of increased activity observed 2–4 h after prolactin treatment (Selmanoff 1985), and a delayed component seen ∼12 h after prolactin treatment (Demarest *et al*. 1984*a*, 1986). More recent electrophysiological data has demonstrated even more rapid actions of prolactin on the electrical activity of TIDA neurons in mice (Brown *et al*. 2012, Romano *et al*. 2013) or rats (Lyons *et al*. 2012). These studies show that prolactin induces a fourfold increase in firing rate within seconds to minutes of application, acutely changing the firing pattern from a basal phasic pattern to a tonically active pattern. Hence, there seem to be multiple mechanisms of prolactin regulation of these dopamine neurons mediated over different time courses.

It was observed that prolactin feedback was markedly impaired in mice lacking the transcription factor STAT5b (Grattan *et al*. 2001), likely through impairment in the long-term regulation of expression of the rate limiting enzyme in dopamine synthesis, tyrosine hydroxylase (Arbogast & Voogt 1991*a*, Ma *et al*. 2005). While such an effect might account for the ‘delayed’ component of prolactin feedback, which requires protein synthesis (Johnston *et al*. 1980), it is unlikely to account for more rapid components of short look feedback. The very rapid action revealed by electrophysiology appears to involve two components: a low voltage component from transient receptor potential (TRP)-like current and high voltage component from inhibition of a Ca^2+^-dependent BK-type K^+^ current, with the latter component being wortmannin sensitive, suggesting an involvement of the PI3K pathway (Lyons *et al*. 2012). The slower component, originally described as ‘rapid’ in neurochemical experiments, with a time course of minutes to hours, likely involves prolactin-induced serine phosphorylation of tyrosine hydroxylase (Ma *et al*. 2005), resulting in increased enzyme activity (Arbogast & Voogt 1997). Together, these three layers of prolactin regulation of hypothalamic dopamine neurons provides a tight homeostatic control, with prolactin rapidly increasing the firing rate of these neurons to induce increased dopamine secretion into the portal blood and rapid suppression of further prolactin secretion from the lactotroph. At the same time, slower but more prolonged changes in tyrosine hydroxylase phosphorylation and transcriptional events to maintain changes in tyrosine hydroxylase gene transcription serve to regulate neuronal function over a much longer time-course, priming the neurons for continued responses to changes in prolactin levels (Grattan & LeTissier 2015).

For endogenous prolactin to function in the short-loop feedback manner, previously described, one important consideration is how this relatively large (197–199 amino acids; 21 kDa) polypeptide hormone crosses the blood brain barrier to gain access to the dopamine neurons. While it is possible that the arcuate nucleus/median eminence region may have an incomplete blood-brain barrier such that hormones can directly access to neurons in this area (Schaeffer *et al*. 2013), it seems unlikely that this is the major mechanism by which prolactin regulates the hypothalamic dopamine neurons. Indeed, systemic administration of prolactin has been shown to simultaneously activate neurons throughout the hypothalamus (Brown *et al*. 2010, Sapsford *et al*. 2012), not simply in the arcuate nucleus. There is clear evidence that systemic prolactin crosses the blood brain barrier through a saturable, carrier-mediated transport system (Walsh *et al*. 1987). As a result, prolactin levels in the cerebrospinal fluid parallel changes in prolactin in the peripheral circulation (Login & MacLeod 1977, Nicholson *et al*. 1980, Grattan & Averill 1991). Because of the high levels of prolactin receptor expression and prolactin binding seen in the choroid plexus (Walsh *et al*. 1978, 1990, Pi & Grattan 1998*a*,*b*, Augustine *et al*. 2003), it has been widely assumed that the prolactin receptor might mediate prolactin entry into the cerebrospinal fluid. However, we have recently shown that prolactin transport into the brain is independent of the prolactin receptor, occurring just as well in prolactin receptor knockout mice (Brown RSE, Wyatt AK, Herbison RE, Knowles PJ, Ladyman SR, Binart N, Banks WA & Grattan DR, unpublished observations). Hence, precise mechanism that translocates prolactin from the blood into the CSF remains to be determined.

### Plasticity in prolactin feedback during lactation

**Figure 2:**
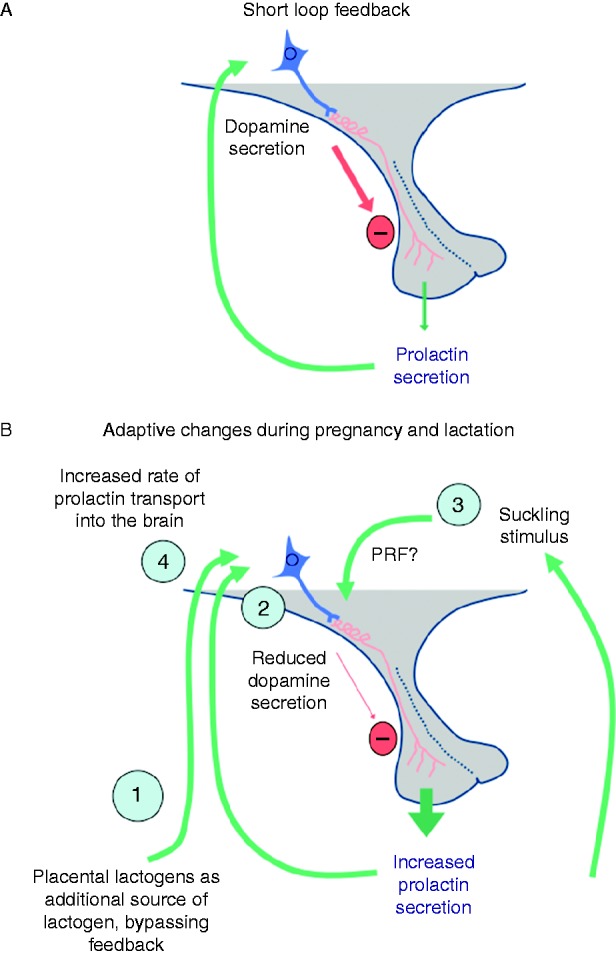
(A) Diagrammatic representation of short loop feedback control of prolactin secretion. (B) Adaptive changes in the regulation of prolactin secretion during pregnancy and lactation. Note that there are multiple adaptive processes to ensure elevated levels of lactogenic hormones present both in the blood and in the brain of the mother, potentially regulating a wide range of functions to facilitate lactation: 1) Production of prolactin-like molecules from the placenta to bypass feedback regulation of pituitary prolactin secretion. 2) Plasticity in the TIDA neuronal response to prolactin, with reduced secretion of dopamine and induction of enkephalin expression. 3) Maternal behavioural adaptation to suckle the pups, providing the most powerful prolactin-releasing stimulus known. 4) Increased transport of prolactin into the brain during lactation.

In order to support a period of hyperprolactinemia during lactation, and thereby promote the milk production that is essential to this state, there is an apparent loss of sensitivity of the short-loop feedback system during late pregnancy and lactation (Grattan *et al*. 2008). This is a remarkable example of adaptive plasticity within a neuroendocrine control network, allowing a sustained period of high prolactin secretion to be maintained unencumbered by a regulatory feedback pathway (see Fig. 2). The mechanisms mediating this adaptive response are only recently becoming elucidated. Up until late pregnancy in rodents, normal negative feedback regulation of prolactin secretion dominates, as previously described, but high levels of placental lactogens are produced. As placental lactogen binds to and activates prolactin receptors, this mimics prolactin action and bypasses the feedback inhibition to ensure that prolactin responsive functions are highly stimulated at this time. Activity of hypothalamic dopamine neurons is maintained by the presence of placental lactogen, so pituitary prolactin secretion is low. Despite the continued presence of placental lactogens, however, there is a decrease in activity of the dopamine neurons during late pregnancy (Andrews *et al*. 2001) associated with a nocturnal surge in pituitary prolactin secretion immediately before parturition (Grattan & Averill 1990, 1995). Hypothalamic dopamine neurons apparently no longer release dopamine in response to prolactin or placental lactogen at this time, rendering the short-loop negative feedback system functionally inactive (Grattan & Averill 1995, Fliestra & Voogt 1997). This adaptation persists into lactation, and dopamine secretion remains low throughout this period of elevated prolactin secretion (Ben-Jonathan *et al*. 1980, Demarest *et al*. 1983). This is an important adaptation, because prolactin is required for milk production and maternal behaviour at this time.

There is a highly coordinated release of prolactin during lactation, caused by the suckling stimulus. This is associated with a decrease in dopamine turnover in hypothalamic dopamine neurons (Selmanoff & Wise 1981, Demarest *et al*. 1983, Selmanoff & Gregerson 1985) and a profound suppression of tyrosine hydroxylase mRNA levels (Wang *et al*. 1993). The original perception that hypothalamic dopamine neurons show a ‘loss of response’ to prolactin at this time has proved to be incorrect. In fact, prolactin receptor expression in the dopamine neurons is maintained (Kokay & Grattan 2005), and acute electrophysiological responses to prolactin persist in lactation (Romano *et al*. 2013). Downstream of the prolactin receptor, however, there is a change in the cellular response. Serine 40 phosphorylation of tyrosine hydroxylase in the median eminence is decreased (Feher *et al*. 2010, Romano *et al*. 2013), resulting in a reduction in the activity of this enzyme (and a reduction in dopamine synthesis). Strikingly, there is a disconnection between neuronal firing and dopamine release at the median eminence. Even though the electrophysiological response to prolactin is unchanged, there is no longer detectable release of dopamine in the median eminence (Romano *et al*. 2013). Prolactin-induced activation of STAT5b in dopamine neurons is reduced during lactation, potentially mediated by an up-regulation of endogenous inhibitors of STAT signaling, the suppressors of cytokine signaling (SOCS) proteins (Anderson *et al*. 2006*a*,*b*, Steyn *et al*. 2008). At the same time as the loss of dopamine secretion, there is an increase in met-enkephalin expression in the dopamine neurons (Merchenthaler 1993, 1994, Szabo *et al*. 2011), and it seems possible that elevated prolactin may drive this met-enkephalin expression (Nahi & Arbogast 2003). Hence, the neurons essentially change their phenotype, changing from being dopaminergic to enkephalinergic. As they still respond electrophysiologically to prolactin, they may be mediating a completely different function of prolactin in the brain during lactation.

It is interesting to note that while the suckling-induced prolactin secretion could be consistent with the ‘dopamine withdrawal’ or ‘disinhibition’ model of prolactin secretion, previously discussed, the chronic reduction in dopamine output seen during lactation complicates this interpretation. If dopamine production is lost, as implied in the data of Romano *et al*. (2013), how can suckling cause an acute increase in prolactin secretion through dopamine withdrawal? Perhaps this is evidence that a suckling-induced ‘prolactin-releasing factor’ may be involved in stimulating prolactin secretion at this time (see further discussion of prolactin-releasing factors in the following section). It is well established that enkephalin can promote prolactin secretion (Cusan *et al*. 1977), and while most evidence suggests that this effect is mediated centrally through regulation of TIDA neurons, it can also act in the pituitary gland to antagonize dopaminergic inhibition of lactotrophs (Enjalbert *et al*. 1979). Could the lactation-specific release of enkephalin from TIDA neurons be functioning as a prolactin-releasing factor, either in the classical sense, by regulating lactotroph function via the portal blood, or through a more local effect on TIDA neurons within the median eminence? Either way, the idea that prolactin might be promoting its own secretion through the same neurons that normally inhibits its secretion, essentially switching from negative to positive feedback at a time when high levels of prolactin are required, is a provocative one worthy of further investigation.

Some significant insight has been provided by recent advances in mapping the neuronal pathways conveying the sucking stimulus through to specific neuronal populations within the hypothalamus. A direct neuronal pathway is involved, transmitting the somatosensory afferent information from the nipple via the spinal cord to the hypothalamus (Berghorn *et al*. 2001). Recent evidence suggests that there is a direct pathway from the subparafascicular nucleus and posterior thalamus to the ventrolateral arcuate nucleus, possibly connecting with the dynorphin neurons located in this region (Szabo *et al*. 2011). Neurons in this pathway express the peptide tuberoinfundibular peptide of 39 residues (TIP39), and this peptide may be a critical regulator of prolactin secretion in response to suckling (Cservenak *et al*. 2010, Dobolyi 2011), acting through the parathyroid hormone 2 receptor (Dobolyi *et al*. 2012) to suppress activity of TIDA neurons.

### Role of a ‘prolactin-releasing factor’?

Ever since Harris' first proposal of the humeral control of the anterior pituitary gland, researchers have searched for a ‘prolactin-releasing factor’ to match that of other pituitary hormones. There have been some notable discoveries, but to date, convincing evidence for a physiological prolactin-releasing factor has not been forthcoming (for reviews, see Freeman *et al*. (2000), Ben-Jonathan & Hnasko (2001) and Crowley (2015)). Most of the factors that regulate prolactin secretion do so by directly or indirectly influencing dopamine secretion from the hypothalamic dopamine neurons. The best example of this is prolactin itself, which stimulates dopamine release to inhibit its own secretion (previously discussed). Other examples are opioid peptides, which are potent stimulators of prolactin secretion, and act predominantly through an inhibition of dopamine neurons. Alternatively, factors may stimulate prolactin secretion through an action on the pituitary gland. The ovarian steroid, estradiol, is an excellent example, acting on lactotrophs to increase prolactin gene expression and increase levels of prolactin released in response to other stimuli (Fink 1988). Neither of these actions would classify as a hypophysiotrophic ‘prolactin releasing factor’, as defined by Harris. For this, the factor would need to be produced in the hypothalamus, be secreted into the portal blood, and act in the pituitary gland to stimulate prolactin secretion (and thereby oppose the actions of the hypothalamic inhibitory hormone).

The levels of prolactin achieved by administration of dopamine antagonists are as high as one would normally associate with prolactin release stimulated under physiological conditions, such as in response to the suckling stimulus (Andrews & Grattan 2004). Thus, it would seem possible to account for most stimulated prolactin secretion simply by a process of disinhibition, removing the normal dopaminergic inhibitory control. However, whether such a total withdrawal of dopamine ever occurs *in vivo* is unlikely (Martinez de la Escalera & Weiner 1992). There is continued interest in the possibility that a physiological ‘prolactin-releasing factor’ exists. Vasoactive intestinal polypeptide (VIP) may be the ancestral regulator of prolactin secretion, since it is the primary ‘prolactin-releasing factor’ in non-mammalian vertebrates (Horseman 1995), and it has stimulatory effects on prolactin secretion in mammals (Murai *et al*. 1989). However, while produced in both the hypothalamus and in the pituitary gland, it is unlikely that VIP acts as a hypophysiotrophic releasing factor, in the sense defined by Harris. It does not appear to be secreted into the portal system at levels higher than in the systemic circulation, nor is it present at elevated levels in the blood at all times that prolactin secretion is high. The same is probably true for a large number of factors that have been investigated as putative ‘prolactin releasing hormones’, including thyrotropin-releasing hormone, oxytocin, galanin, salsolinol, prolactin-releasing peptide and others (reviewed in Freeman *et al*. (2000), Crowley (2015) and Grattan & LeTissier (2015)). Many of these factors may influence prolactin secretion, either from effects on hypothalamic dopamine neurons, or from effects on pituitary lactotrophs, but none have proven to meet the criteria to be considered hypothalamic hypophysiotrophic factors.

Freeman's group tackled this question by investigating whether changes in prolactin could be observed independently of dopaminergic inhibition. They observed that administration of the D2 antagonist domperidone induced different levels of prolactin secretion at different times of the day (Arey *et al*. 1989). Assuming that antagonism of dopamine was complete at each time point, they interpreted these data to demonstrate the existence of an ‘endogenous stimulatory rhythm’, where factors from the hypothalamus (including oxytocin and VIP) were promoting prolactin secretion at specific times in dependently of dopamine (Arey & Freeman 1990, 1992*a*,*b*), but this stimulation was usually masked by the prevailing dopaminergic tone. Importantly, they also showed that endogenous stimuli that reduce dopamine input to the pituitary, such as suckling, could also reveal this stimulatory rhythm, promoting different levels of prolactin secretion at different times of the day (Arey *et al*. 1991). These data provide convincing evidence of dopamine-independent regulation of prolactin secretion, but cannot conclusively prove it is mediated by a ‘prolactin-releasing factor’. An alternative possibility is that endogenous circadian regulators within the pituitary gland might influence that amount of prolactin released at different times of the day (Becquet *et al*. 2014). Circadian regulation of prolactin secretion has also been documented via melatonin actions in the pars tuberalis (Lincoln *et al*. 2003).

If there is a physiological ‘prolactin-releasing factor’, it remains elusive. One possible reason for this is that the stimulatory control exerted from the hypothalamus may not be a ‘classical’ system, as defined by Harris. In the late 1980s and early 1990s, there was a particularly well-developed story regarding a putative prolactin-releasing factor coming from a subpopulation of melanotrophs in the intermediate lobe of the pituitary, as opposed to the median eminence. This factor was originally discovered by Murai & Ben-Jonathan (1987, 1990), who showed that surgical removal of the posterior pituitary (including the intermediate lobe) impaired the prolactin release in response to estradiol administration or suckling. This was subsequently supported by a number of other groups, with studies demonstrating reduced or absent prolactin secretion after removal of the neurointermediate lobe (Samson *et al*. 1990, Averill *et al*. 1991, Andrews & Grattan 2004). Moreover, mice with secretory tumours of the intermediate lobe were hyperprolactinemic (Allen *et al*. 1995). Despite extensive effort at characterisation, particularly from the Ben-Jonathan group, the specific identity of this factor (or factors) has not been identified, although a number of known prolactin secretagogues (TRH, oxytocin, VIP) were excluded (Allen *et al*. 1995, Hnasko *et al*. 1997). Nevertheless, this highlights the possibility that ‘non classical’ prolactin releasing systems may remain to be discovered.

Based on the magnitude of prolactin release in response to dopamine inhibition (Andrews & Grattan 2004), I have previously held the view that most prolactin release could be accounted for by a decrease in dopamine. Our new data showing that even after marked reductions in dopamine secretion during lactation in mice (Romano *et al*. 2013), there is a sustained ability to regulate prolactin secretion in response to suckling, however, has forced a re-think of this view. It would seem that other regulatory factors must be involved in the physiological regulation of prolactin secretion, particularly during lactation when it is most required. Whether one or more of these factors becomes the long sought ‘prolactin-releasing factor’ predicted by Harris remains an exciting area needing further investigation.

### Regulation of prolactin secretion by estradiol

The ovarian steroid estradiol is arguably one of the most important regulators of prolactin secretion in several different physiological states. In the pituitary gland, estradiol is a major stimulator of prolactin secretion, although this is principally through a classical genomic regulation of prolactin gene expression (Lieberman *et al*. 1981), by increasing the number of lactotrophs (Takahashi *et al*. 1984, Scully *et al*. 1997, Kansra *et al*. 2005, 2010, Nolan & Levy 2009) and by modifying lactotroph responsiveness to other regulators (De Lean & Labrie 1977, Raymond *et al*. 1978, West & Dannies 1980), although a rapid non-genomic actions of estradiol stimulating prolactin secretion have been described (Huerta-Ocampo *et al*. 2005). The TIDA neurons also express receptors for both estradiol (estrogen receptor alpha (ERα)) and progesterone (Sar 1984, 1988, Fox *et al*. 1990, Lonstein & Blaustein 2004, Steyn *et al*. 2007), and gonadal steroids regulate prolactin secretion indirectly through actions on these neurons (Jones & Naftolin 1990, Arbogast & Voogt 1993, 1994, DeMaria *et al*. 2000). These actions of estradiol are particularly important during the reproductive cycle and during pregnancy. The predominant direct action of estradiol on TIDA neurons is one of inhibition (Demarest *et al*. 1984*b*, Arita & Kimura 1987, DeMaria *et al*. 2000, Morel *et al*. 2009), suppressing TH expression (Blum *et al*. 1987, Morrell *et al*. 1989) and activity (Pasqualini *et al*. 1993), and reducing secretion of dopamine into the portal blood (Cramer *et al*. 1979), thereby facilitating prolactin secretion. The estradiol-induced proestrous prolactin surge is associated with a steroid-dependent decline in TIDA activity (DeMaria *et al*. 1998, Yen & Pan 1998, Liu & Arbogast 2008, 2010), with a prominent role for progesterone suppressing dopamine release during the plateau phase of the surge (Arbogast & Ben-Jonathan 1990, Arbogast & Voogt 1994). Similarly, ovarian steroids play a critical role in controlling the twice-daily prolactin surges required to sustain luteal function pregnancy in rodents (Gunnet & Freeman 1983, Arbogast & Voogt 1991*b*). The rising levels of estradiol during pregnancy are also critical to promoting prolactin secretion, particularly during late pregnancy (Grattan & Averill 1990, Andrews 2005), and to the plasticity in the TIDA neurons as previously described (Grattan *et al*. 2008). In addition to regulation of prolactin secretion, estradiol may also regulate the cellular responses to prolactin. In the brain, many of the neurons expressing the prolactin receptor also express ERα (Furigo *et al*. 2014). Estradiol may regulate prolactin receptor expression on neurons (Lerant & Freeman 1998), and several of the actions of prolactin are dependent on the presence of estradiol (Anderson *et al*. 2008). Thus, estradiol acts at multiple levels to both directly and indirectly regulate prolactin synthesis, secretion and action.

## Neuroendocrine functions of prolactin

Prolactin was identified and named for its critical role in the physiology of lactation, and this remains its best-characterised function. Prolactin is indispensible for lactation. However, a vast array of additional functions has also been characterised. These have been thoroughly reviewed by Paul Kelly's group (Bole-Feysot *et al*. 1998), who classified the functions under six broad headings: water and electrolyte balance, growth and development, endocrinology and metabolism, brain and behaviour, reproduction and immune regulation and protection. The breadth of potential functions is astounding and difficult to conceptualise into a theoretical framework. Many of the reported actions of prolactin appear to be redundant, as evidenced by the lack of a significant phenotype in the prolactin or prolactin-receptor knockout mice. This may simply be the evolutionary result of a phylogenically old signalling molecule being used for multiple adaptive roles in homeostasis. One can see examples where prolactin has subserved similar functions across many species. For example, in fish and amphibia, it is involved in electrolyte balance, and movement of ions and water across epithelial barriers (Bole-Feysot *et al*. 1998). Perhaps this is not so different from inducing secretion of nutrients and electrolytes from an epithelial gland, as in the crop milk of pigeons, and the breast milk of mammals. Similarly, prolactin has been implicated in parental behaviour ranging from nest fanning in fish, through to incubation and brooding behaviour in birds, to lactation and maternal behaviour in mammals. When viewed from an evolutionary context, it seems logical that the nurturing parental behaviour actions of prolactin might have evolved in parallel with a nutrient synthesis and secretion role, providing an adaptive advantage of novel reproductive strategies.

There is insufficient space in this review to do justice to the wide range of potential prolactin-sensitive functions. Instead, I have taken the strategy of focusing on functions that are specifically regulated when endogenous prolactin levels are high. Apart from the estrogen-induced prolactin surge during the female reproductive cycle (which may not occur in all species (Ben-Jonathan *et al*. 2008), and the response to stress, which is transient and of low magnitude (Gala 1990), prolactin levels are typically maintained at low levels as a consequence of the highly effective short loop negative feedback. The exception to this is pregnancy and lactation, where mammals exhibit at least three adaptations to ensure high levels of lactogenic hormone activity throughout these conditions (see Fig. 2). First, there is the production of placental lactogen and/or decidual prolactin, lactogens from reproductive tissues. These hormones act on the prolactin receptor, and therefore bypass the short-loop feedback regulation of the anterior pituitary gland to provide constantly elevated levels of lactogenic hormones throughout pregnancy. Secondly, there are the adaptive changes in feedback occurring in the maternal hypothalamic dopamine neurons, previously discussed, changing the manner in which they respond to prolactin, enabling high secretion to prolactin to be maintained from the maternal pituitary after the pregnancy-specific placental lactogens are lost at parturition. Thirdly, there is the hormone-dependent expression of maternal behavior, with the consequent suckling stimulus from the pups providing the most powerful stimulus to pituitary prolactin secretion that is known in mammals. As previously discussed, this might involve chronic and/or acute dopamine withdrawal, as well as the additional stimulus of an as yet unidentified ‘prolactin-releasing factor’ to maintain elevated levels of prolactin. Finally, we have recent evidence suggesting that transport of prolactin into the brain is increased during lactation (Brown RSE, Wyatt AK, Herbison RE, Knowles PJ, Ladyman SR, Binart N, Banks WA & Grattan DR, unpublished observations), suggesting that many of the CNS functions of prolactin might be further enhanced at this time. These multiple adaptive changes make a compelling argument to focus on pregnancy and lactation as the most critical time for prolactin actions in the body.

It is absolutely clear that these elevated levels of lactogenic hormones are required for development of the mammary gland during pregnancy and for milk production during lactation. The regulation of mammary function by prolactin is extensively reviewed elsewhere (Hovey *et al*. 2001, Trott *et al*. 2012). It is important to recognize, however, that prolactin and placental lactogen are also able to act in a wide variety of other tissues in the body. The prolactin receptor is widely expressed in numerous body systems, including bone, adipose tissue, gut, reproductive tract, skin, immune system, pituitary and brain (Bole-Feysot *et al*. 1998, Goffin *et al*. 2002), and hence, when prolactin is elevated there is potential for a wide variety of systems to be influenced. In recent reviews (Grattan & Kokay 2008, Grattan & LeTissier 2015), we have proposed the hypothesis that the wide range of potential actions of prolactin in the body make some collective sense if considered within the context of the physiological hyperprolactinemic state of pregnancy and lactation. These are complex and demanding processes for a mother, requiring multiple diverse systems to undergo adaptive changes to facilitate her successful transition from the non-pregnant into the maternal state. A selection of these adaptive changes, and a summary of the evidence that prolactin might influence the adaptive response, are outlined in Table 1. Here, prolactin can be considered as interoceptive sensory information for the body, informing it of its new physiological state. The changes it induces are adaptive, which means that the functions are unlikely to completely fail in the absence of prolactin action, but they might not perform optimally. This would account for the absence of widespread adverse phenotype in the prolactin receptor knockout mice. It is appropriate to point out that many of the associations shown in Table 1 are, at this stage, correlative only, and comprehensive investigation proving that prolactin may be mediating a particular adaptive change will require significant further work. Nevertheless, we have found this to be a useful construct for understanding why prolactin might be influencing such a wide range of biological function. We used to be concerned by the question of, ‘Why would there be over 300 physiological actions of prolactin?’ Now, we can consider each of the different tissues that express the prolactin receptor and ask the question, ‘Why might this tissue need to change its function during lactation?’

Comprehensive reviews of the wide range of actions of prolactin in the body are available elsewhere (Bole-Feysot *et al*. 1998, Freeman *et al*. 2000), as is our hypothesis regarding the role of prolactin in the physiological adaptation to pregnancy (Grattan & Kokay 2008, Grattan & LeTissier 2015), and hence, I will not go into detail here. In the final section of this review, I would like to briefly highlight three selected examples from recent research. The first of these, the metabolic functions of prolactin, nicely illustrates the context previously outlined that prolactin is acting in a number of different tissues and cell types in the mother to facilitate adaptation to pregnancy or lactation. The second example, looking at prolactin effects on fertility, highlights how functions of prolactin that might be considered adaptive in a lactating female, might be maladaptive should high prolactin occur at an inappropriate time. The third example asks the question, ‘What is prolactin doing in the male?’ This example is used to acknowledge the fact that some of the known functions of prolactin do not comfortably fit into the conceptual framework previously presented.

### Metabolic function of prolactin

This is, perhaps, the best example of the pleiotropic role of prolactin (defined to mean a single gene product, prolactin, exerting multiple seemingly diverse actions). Prolactin receptors are expressed on multiple tissues involved in metabolic regulation, including adipose tissue, liver, pancreas and the brain. It appears to play a broad role in both pancreatic and adipose development. In adipose tissue, prolactin is essential in adipogenesis and adipocyte differentiation, as well as modulating lipid metabolism. It also regulates the secretion is several adipokines, including stimulation of leptin and inhibition of adiponectin production (Ben-Jonathan *et al*. 2006, Carre & Binart 2014). In the pancreas, it promotes growth of islets during development (Freemark *et al*. 2002), and increases insulin expression and glucose-stimulated insulin secretion (Sinha & Sorenson 1993, Brelje *et al*. 1994, 2004). It also increases expression of glucose transporter 2 and promotes glucose entry into the β-cells (Petryk *et al*. 2000), resulting in enhanced activity of glucose-sensitive enzymes such as glucokinase (Weinhaus *et al*. 2007). In both adipose tissue and pancreas, these actions are likely to be profoundly important during pregnancy and lactation. Lipid metabolism is altered, with lipid mobilisation from stores and utilisation in mamary gland promoted by prolactin (Barber *et al*. 1992). Adaptive changes in glucose homeostasis are also important in pregnancy (Rieck & Kaestner 2010). Maternal tissues develop insulin resistance to preferentially direct glucose to the fetal/placental compartment (Herrera 2000), and to ensure the maternal tissues continue to receive the nutrients required, there is increased demand for maternal insulin secretion, and glucose-stimulated insulin secretion increases. To adapt to this altered demand, there is significant proliferation of β-cells in the islets (Parsons *et al*. 1992), enhanced insulin synthesis (Bone & Taylor 1976), and decreased threshold for glucose-stimulated insulin secretion (Sorenson & Parsons 1985), with prolactin playing a critical adaptive role in promoting these changes (Newbern & Freemark 2011). Failure of this adaptive response results in gestational diabetes (Ramos-Roman 2011).

These peripheral actions of prolactin on metabolism are complemented by CNS actions of prolactin to promote appetite and potentially regulate glucose homeostasis. Systemic prolactin administration increases food intake in a variety of species (Moore *et al*. 1986, Gerardo-Gettens *et al*. 1989, Noel & Woodside 1993, Buntin *et al*. 1999), independent of potential effects on ovarian steroids (Noel & Woodside 1993, 2007, Sauvé & Woodside 1996). Thus, the elevated prolactin secretion is likely to contribute to the rapid increase in food intake during pregnancy (Shirley 1984, Ladyman & Grattan 2004, Ladyman *et al*. 2012) and the extreme hyperphagia of lactation (Woodside 2007, Woodside *et al*. 2012). Prolactin also induces functional leptin resistance, which would contribute to increased food intake (Naef & Woodside 2007, Augustine & Grattan 2008), potentially mediating the well-established leptin resistance of pregnancy (Grattan *et al*. 2007*a*, Augustine *et al*. 2008, Ladyman 2008, Ladyman *et al*. 2010). Prolactin receptors are found in many of the nuclei involved in the homeostatic regulation of food intake, including the arcuate, ventromedial and paraventricular nuclei (Bakowska & Morrell 1997, Pi & Grattan 1998*b*, Brown *et al*. 2010). However, prolactin receptors do not appear to be expressed in the arcuate neuropeptide Y (NPY) and proopiomelanocortin (POMC) neurons (Li *et al*. 1999, Chen & Smith 2004, Kokay & Grattan 2005) that regulate appetite. Hence, it seems likely that prolactin acts downstream of the arcuate neurons, such as at the paraventricular nucleus. Consistent with this hypothesis, localised injections of prolactin directly into the paraventricular nucleus stimulate food intake in a dose-dependent manner in female rats (Sauvé & Woodside 2000).

Thus, seemingly diverse actions of prolactin in multiple different cell types can be unified into a single adaptive function, which is metabolic adaptation to pregnancy, increasing energy availability to the mother and offspring. This is an example of the conceptual framework previously outlined, and it can be viewed as a positive, adaptive mechanism. Should hyperprolactinemia occur at an inappropriate time, however, then one might predict this could contribute to metabolic disorders. There is some evidence for this. While it is not universally observed, patients with hyperprolactinemia are prone to excessive weight gain (Creemers *et al*. 1991, Delgrange *et al*. 1999, Doknic *et al*. 2002, Baptista *et al*. 2004), and normalisation of prolactin levels using dopamine agonists is associated with weight loss (Greenman *et al*. 1998, Doknic *et al*. 2002, Galluzzi *et al*. 2005). Interestingly, genome-wide association studies have revealed that a common variant adjacent to the prolactin gene is associated with obesity (Meyre *et al*. 2009, Nilsson *et al*. 2011) suggesting that abnormalities in prolactin or prolactin signalling may contribute to human obesity.

### Hyperprolactinemia and infertility

Hyperprolactinemia causes infertility in both males and females (Patel & Bamigboye 2007), and this provides an even more clear-cut example of a potentially adaptive function under certain conditions becoming clearly maladaptive in another situation. The mechanism by which prolactin inhibits the reproductive axis is not clear, but evidence suggests that prolactin impacts fertility through actions on GnRH neurons. In humans, hyperprolactinaemia is associated with a marked reduction in both the frequency and amplitude of LH pulses (Bohnet *et al*. 1976, Matsuzaki *et al*. 1994) indicative of a change in GnRH pulses, and the suppression of LH pulsatility can be reversed by reducing serum prolactin concentrations to normal (Moult *et al*. 1982). While prolactin could exert effects in either the pituitary or gonad, pulsatile GnRH replacement can reverse the infertility induced by hyperprolactinaemia (Polson *et al*. 1986, Matsuzaki *et al*. 1994, Lecomte *et al*. 1997), suggesting a prolactin-induced suppression of GnRH release is the proximal cause of infertility. Similarly, prolactin suppresses both the frequency and amplitude of LH pulses in male and female rats (Cohen-Becker *et al*. 1986, Fox *et al*. 1987, Park & Selmanoff 1991, Park *et al*. 1993) and measurements of GnRH secretion into the portal blood have revealed prolactin-induced suppression of GnRH release (Weber *et al*. 1983, Koike *et al*. 1984, 1991, Sarkar *et al*. 1992). Furthermore, hyperprolactinaemia has been shown to prevent the castration-induced increase in GnRH mRNA expression in rats (Selmanoff *et al*. 1991). Thus, although there is ample evidence that prolactin can act in the pituitary gland to suppress LH secretion (Smith 1978, 1982, Cheung 1983, Morel *et al*. 1994, Tortonese *et al*. 1998), in animal models, as in clinical studies, the primary cause of infertility appears to be the suppression of the activity of GnRH neurons. This effect of prolactin is unlikely to be mediated directly by an action on GnRH neurons, as the majority of these neurons do not express the prolactin receptor (Grattan *et al*. 2007*b*, Kokay *et al*. 2011). Thus, prolactin-induced inhibition of GnRH neurons must involve prolactin-sensitive afferents to these cells. Interestingly, most prolactin responsive neurons also express ERα (Furigo *et al*. 2014), so prolactin may share a common mechanism of regulating GnRH with the negative feedback pathway mediated by estradiol. As such, kisspeptin neurons have emerged as the most likely intermediate regulators.

Since first being identified as essential for puberty in humans (de Roux *et al*. 2003, Seminara *et al*. 2003), kisspeptin neurons are now recognised as critical parts of the circuit regulating activity of the GnRH neurons that control fertility (Pinilla *et al*. 2012). Kisspeptin neurons may have an important role in mediating the suppressive effect of prolactin on fertility. Kisspeptin is the most potent stimulator of GnRH neuronal activity yet identified (Han *et al*. 2005, Liu *et al*. 2008). In most mammalian species, there are two populations of kisspeptin neurons, with kisspeptin neurons in the rostral periventricular area of the third ventricle (RP3V) playing an essential role in enabling ovulation in rodents by activating GnRH neurons (Herbison 2008, Clarkson & Herbison 2009, Oakley *et al*. 2009), while kisspeptin neurons in the arcuate nucleus are thought to be involved in the regulation of the basal pulsatile secretion of GnRH (Li *et al*. 2009, Roseweir *et al*. 2009, Lehman *et al*. 2010, Navarro *et al*. 2011). Prolactin receptors are expressed in the majority of kisspeptin neurons in both populations (Kokay *et al*. 2011, Li *et al*. 2011), and prolactin has recently been shown to induce the phosphorylation of signal transducer and activator of transcription 5 (pSTAT5) in arcuate nucleus kisspeptin neurons in the rat (Araujo-Lopes *et al*. 2014) and rostral hypothalamic kisspeptin neurons in the mouse (Brown *et al*. 2014). Chronic infusion of prolactin in female mice abolished estrous cyclicity and suppressed global *Gnrh* and *Kiss1* mRNA expression in the hypothalamus, while kisspeptin therapy restored estrous cycles in hyperprolactinemic mice (Sonigo *et al*. 2012), consistent with the hypothesis that prolactin-induced suppression of GnRH secretion is mediated by an inhibition of kisspeptin neurons.

Clearly, under most conditions, hyperprolactinemia represents a pathological condition with adverse consequences. During pregnancy and lactation, however, hyperprolactinemia is physiologically appropriate. When viewed from this context, an inhibitory action of prolactin on fertility during pregnancy and lactation would be highly adaptive, allowing the mother to focus energy on feeding her offspring, before investing resources in a further pregnancy (Valeggia & Ellison 2009). Lactation is associated with a period of infertility in most mammalian females, including women (McNeilly 2001*a*). In humans, this function serves as a critical regulator of population growth, spacing the timing of births to allow the mother to ration her metabolic investment across sequential pregnancies (Short 1976). Despite the extensive impact on mammalian reproductive physiology, our understanding of the mechanisms mediating lactational infertility remains incomplete (McNeilly 1994, 2001*a*,*b*). It is clear that suckling is the critical inhibitory signal (Tsukamura & Maeda 2001), and the principle cause of infertility is an almost-complete suppression of the pulsatile secretion of GnRH from the hypothalamus, the consequent loss of pituitary gonadotropin secretion and ovulation failure (Fox & Smith 1984). The pathways linking the suckling stimulus to the suppression of ovulation, however, are unclear. *Kiss1* mRNA and protein levels are reduced in the arcuate nucleus of lactating rats associated with the suppression of pulsatile GnRH secretion during lactation (Yamada *et al*. 2007, 2012, Smith *et al*. 2010, True *et al*. 2011, Araujo-Lopes *et al*. 2014, Ladyman & Woodside 2014), and in both populations in the mouse (Brown *et al*. 2014). The RP3V kisspeptin population is harder to study in rats (Yamada *et al*. 2007, Desroziers *et al*. 2010), with reports of both *Kiss1* mRNA remaining unchanged during lactation (Yamada *et al*. 2007) and of kisspeptin protein increasing while *Kiss1* mRNA labeling decreased during lactation (Smith *et al*. 2010). More importantly, we have also shown that the reduction in kisspeptin expression results in complete loss of capacity for these neurons to activate GnRH neurons, even if they are stimulated exogenously (Liu *et al*. 2014). Given the similarity between the effect of suckling and the effect of prolactin, and the knowledge that suckling stimulates prolactin secretion, it seems likely that elevated prolactin during pregnancy and lactation contributes to the infertility of lactation, but this remains to be proven, and the relative roles of prolactin and/or suckling may be different in different species.

### What does prolactin do in the male?

The hypothesis that most prolactin actions in the body can be tied to the adaptation to pregnancy and lactation clearly does not explain effects of prolactin in the male. Up to 40% of the male pituitary gland is dedicated to lactotrophs, suggesting that some function is retained in males, but knockout studies have not identified an essential function of prolactin. While there is no male equivalent of lactation, many of the other functions of prolactin in females can also be observed in males. For example, as in females, prolactin seems to be involved in parental behaviour in males, although the relative role that the mammalian father plays in parental care of offspring varies amongst species. In species where the male plays some role in rearing of the offspring, including humans (Gordon *et al*. 2010, Gettler *et al*. 2012), studies have found a association between prolactin and paternal care (Schradin & Anzenberger 1999), although the overall picture is unclear and controversial (Schradin 2007, Wynne-Edwards & Timonin 2007). Nevertheless, paternal recognition of offspring is consistent amongst most species. Pup-contact by male rats can lead to some forms of parental care behaviour and this is associated with an increase in serum prolactin, as well as increased expression of the long-form of the prolactin receptor in the brain (Sakaguchi *et al*. 1996). Further evidence for a direct role for prolactin in paternal recognition of offspring has been shown by studies of *Prlr*^*−/−*^ fathers, who fail to distinguish adult offspring from non-offspring, possibly as a result of failure of prolactin-induced neurogensis in the sub-ventricular zone and the dentate gyrus (Mak & Weiss 2010).

As in females, pathological hyperprolactinemia causes infertility in males, but it is not clear that there is an adaptive role for prolactin in male reproduction. At lower levels, prolactin contributes a range of functions in the male reproductive tract, revealed by subtle reproductive deficits in the prolactin receptor deficient mice (Grattan & LeTissier 2015). In addition, many of the metabolic and immune functions of prolactin can be observed in males, but whether prolactin levels are ever sufficient for these effects to be of physiological significance is uncertain. Perhaps the most consistent stimulus for prolactin secretion in males is stress, but the functional consequences of this response are not well-understood (Gala 1990).

## Conclusion

While Harris was correct in proposing that the brain controls prolactin secretion, the hypothalamo-prolactin axis proved itself to be quite different from all other pituitary systems. It remains the most complex and versatile of all of the hypothalamo-pituitary axes. Even if we just consider the relatively simple task of controlling milk production during lactation, there is much that remains to be understood, such as the possible role of one or more prolactin-releasing factors during the suckling stimulus, and the mechanism controlling the loss of dopamine production in the TIDA neurons and the changes in prolactin negative feedback. If we include the wide range of additional functions of prolactin, then the complexity becomes overwhelming. I have presented here a context to attempt to understand the pleiotropic roles of prolactin, arguing that many of the functions of prolactin can be unified into the overall task of maternal adaptation to pregnancy and lactation. Within this context, prolactin function promotes adaptive changes in a variety of body systems, but such actions can also be maladaptive, in a different context, if hyperprolactinemia occurs at an inappropriate time. This theoretical construct presents many new opportunities for generating testable hypotheses about prolactin function. But there are also many functions that do not fit easily into this construct, providing further opportunities for expanding our understanding. I anticipate that the coming availability of novel tools for investigating prolactin function, including gene-targeting approaches that allow conditional regulation of prolactin responsive cells, will provide the impetus for a new wave of research to enhance our understanding of this fascinating system. Sixty years on from Geoffrey Harris' prescient predictions, we still have a lot of work to do to understand the hypothalamo-prolactin system.

## Footnote

This paper is part of a thematic review section on 60 years of neuroendocrinology. The Guest Editors for this section were Ashley Grossman and Clive Coen.

## Figures and Tables

**Table 1:** Role of prolactin in the maternal adaptation to pregnancy

**Tissue or function**	**Adaptive change during pregnancy**	**Evidence for possible role of prolactin?**	**Selected references**
Mammary gland/lactation	Branching and alveolar development of mammary gland	Unequivocal role, with lactation lost in Prlr−/− mice	Ormandy *et al*. (1997) and Trott *et al*. (2012)
Milk secretion
Maternal behaviour	Retrieval and nursing of pups	Prolactin advances maternal responsiveness to pups in rats	Rosenblatt (1967), Bridges *et al*. (1985, 1990, 1996) and Bridges & Ronsheim (1990)
Impairs behaviour in Prlr−/− mice	Lucas *et al*. (1998)
Adult neurogenesis	Increased neurogenesis in the subventricular zone of the maternal brain	Driven by prolactin changes of pregnancy	Shingo *et al*. (2003)
Important for mood and behavioural changes *post partum*	Larsen *et al*. (2008) and Larsen & Grattan (2010, 2012)
Pancreatic β cell/glucose homeostasis	Maternal tissues become insulin resistant to promote glucose transfer to fetus	Prolactin receptors expressed in beta cells, and expression increased during pregnancy	Moldrup *et al*. (1993), Sorenson & Stout (1995) and Brelje *et al*. (2004)
	Expansion of beta cells in mother to increase insulin production, to prevent gestational diabetes	Prolactin stimulates insulin expression, secretion and beta cell proliferation	Karnik *et al*. (2007)
Impaired glucose tolerance during pregnancy in Prlr+/− mice	Huang *et al*. (2009), Kim *et al*. (2010), Rieck & Kaestner (2010), Schraenen *et al*. (2010) and Zhang *et al*. (2010)
Appetite regulation	Increased appetite and development of leptin resistance	Prolactin stimulates food intake in non-pregnant animals	Gerardo-Gettens *et al*. (1989), Sauvé & Woodside (1996, 2000) and Naef & Woodside (2007)
	Fat deposition during pregnancy, mobilisation during lactation	Contributes to the development of leptin resistance during pregnancy	Augustine & Grattan (2008), Ladyman (2008) and Ladyman *et al*. (2010)
Prolactin contributes to appetite drive during lactation	Woodside *et al*. (2012)
Bone and calcium homeostasis	Increased calcium uptake and mobilisation of calcium stores for fetal skeletal growth and for milk production	Prolactin receptors on osteoblasts and chondrocytes	Charoenphandhu *et al*. (2010) and Wongdee *et al*. (2011)
		Prolactin stimulates bone turnover	
Prolactin promotes calcium absorption in the gut
Reproduction	Maintenance of pregnancy (in rodents).	Prolactin stimulates corpus luteum function in rodents, essential to maintain pregnancy	Gibori & Richards (1978) and Bachelot *et al*. (2009)
	Loss of reproductive cycle during pregnancy, persisting during lactation	Hyperprolactinemia causes infertility	Patel & Bamigboye (2007)
Possible/likely involvement in lactational infertility	McNeilly (2001*a*,*b*) and Liu *et al*. (2014)
Stress responses	Reduced response to stress during late pregnancy and lactation, to minimise exposure of offspring to glucocorticoids	Prolactin is anxiolytic and reduced stress responses in males and non-pregnant females	Shanks *et al*. (1999), Torner *et al*. (2001), Lonstein (2007) and Brunton & Russell (2008)
Role during pregnancy and lactation likely, but unproven	Torner & Neumann (2002) and Slattery & Neumann (2008)
Oxytocin secretion	Marked change in firing pattern, generation of ‘burst’ firing to facilitate parturition and milk ejection	Prolactin receptors on oxytocin neurons, and acute inhibitory effects on activity	Kokay *et al*. (2006) and Sapsford *et al*. (2011)
Stimulation of oxytocin gene expression	Parker *et al*. (1991) and Ghosh & Sladek (1995a,b)
Prolactin secretion	Altered feedback to facilitate prolactin secretion	Prolactin continues to stimulate TIDA neurons, but dopamine secretion is decreased	Grattan *et al*. (2008) and Romano *et al*. (2013)
		Induction of enkephalin production in TIDA	Merchenthaler *et al*. (1994) and Nahi & Arbogast (2003)

## References

[bib1] Allen DL, Low MJ, Allen RG, Ben-Jonathan N (1995). Identification of two classes of prolactin-releasing factors in intermediate lobe tumors from transgenic mice. Endocrinology.

[bib2] Anderson GM, Beijer P, Bang AS, Fenwick MA, Bunn SJ, Grattan DR (2006a). Suppression of prolactin-induced signal transducer and activator of transcription 5b signaling and induction of suppressors of cytokine signaling messenger ribonucleic acid in the hypothalamic arcuate nucleus of the rat during late pregnancy and lactation. Endocrinology.

[bib3] Anderson ST, Barclay JL, Fanning KJ, Kusters DH, Waters MJ, Curlewis JD (2006b). Mechanisms underlying the diminished sensitivity to prolactin negative feedback during lactation: reduced STAT5 signalling and upregulation of cytokine-inducible SH2-domain-containing protein (CIS) expression in tuberoinfundibular dopaminergic neurons. Endocrinology.

[bib4] Anderson GM, Kieser DC, Steyn FJ, Grattan DR (2008). Hypothalamic prolactin receptor messenger ribonucleic acid levels, prolactin signaling, and hyperprolactinemic inhibition of pulsatile luteinizing hormone secretion are dependent on estradiol. Endocrinology.

[bib5] Andrews ZB (2005). Neuroendocrine regulation of prolactin secretion during late pregnancy: easing the transition into lactation. Journal of Neuroendocrinology.

[bib6] Andrews ZB, Grattan DR (2004). The roles of dopamine and the neurointermediate lobe of the pituitary in the regulation of prolactin secretion during late pregnancy in rats. Journal of Neuroendocrinology.

[bib7] Andrews ZB, Kokay IC, Grattan DR (2001). Dissociation of prolactin secretion from tuberoinfundibular dopamine activity in late pregnant rats. Endocrinology.

[bib8] Annunziato L, Moore KE (1978). Prolactin in CSF selectively increases dopamine turnover in the median eminence. Life Sciences.

[bib9] Araujo-Lopes R, Crampton JR, Aquino NS, Miranda RM, Kokay IC, Reis AM, Franci CR, Grattan DR, Szawka RE (2014). Prolactin regulates kisspeptin neurons in the arcuate nucleus to suppress LH secretion in female rats. Endocrinology.

[bib10] Arbogast LA, Ben-Jonathan N (1990). The preovulatory prolactin surge is prolonged by a progesterone-dependent dopaminergic mechanism. Endocrinology.

[bib11] Arbogast LA, Voogt JL (1991a). Hyperprolactinemia increases and hypoprolactinemia decreases tyrosine hydroxylase messenger ribonucleic acid levels in the arcuate nuclei, but not the substantia nigra or zona incerta. Endocrinology.

[bib12] Arbogast LA, Voogt JL (1991b). Mechanisms of tyrosine hydroxylase regulation during pregnancy: evidence for protein dephosphorylation during the prolactin surges. Endocrinology.

[bib13] Arbogast LA, Voogt JL (1993). Progesterone reverses the estradiol-induced decrease in tyrosine hydroxylase mRNA levels in the arcuate nucleus. Neuroendocrinology.

[bib14] Arbogast LA, Voogt JL (1994). Progesterone suppresses tyrosine hydroxylase messenger ribonucleic acid levels in the arcuate nucleus on proestrus. Endocrinology.

[bib15] Arbogast LA, Voogt JL (1997). Prolactin (PRL) receptors are colocalized in dopaminergic neurons in fetal hypothalamic cell cultures: effect of PRL on tyrosine hydroxylase activity. Endocrinology.

[bib16] Arey BJ, Freeman ME (1990). Oxytocin, vasoactive-intestinal peptide, and serotonin regulate the mating-induced surges of prolactin secretion in the rat. Endocrinology.

[bib17] Arey BJ, Freeman ME (1992a). Activity of oxytocinergic neurons in the paraventricular nucleus mirrors the periodicity of the endogenous stimulatory rhythm regulating prolactin secretion. Endocrinology.

[bib18] Arey BJ, Freeman ME (1992b). Activity of vasoactive intestinal peptide and serotonin in the paraventricular nucleus reflects the periodicity of the endogenous stimulatory rhythm regulating prolactin secretion. Endocrinology.

[bib19] Arey BJ, Averill RL, Freeman ME (1989). A sex-specific endogenous stimulatory rhythm regulating prolactin secretion. Endocrinology.

[bib20] Arey BJ, Kanyicska B, Freeman ME (1991). The endogenous stimulatory rhythm regulating prolactin secretion is present in the lactating rat. Neuroendocrinology.

[bib21] Arimura A, Dunn JD, Schally AV (1972). Effect of infusion of hypothalamic extracts on serum prolactin levels in rats treated with nembutal, CNS depressants or bearing hypothalamic lesions. Endocrinology.

[bib22] Arita J, Kimura F (1987). Direct inhibitory effect of long term estradiol treatment on dopamine synthesis in tuberoinfundibular dopaminergic neurons: *in vitro* studies using hypothalamic slices. Endocrinology.

[bib23] Asa SL, Kelly MA, Grandy DK, Low MJ (1999). Pituitary lactotroph adenomas develop after prolonged lactotroph hyperplasia in dopamine D2 receptor-deficient mice. Endocrinology.

[bib24] Augustine RA, Grattan DR (2008). Induction of central leptin resistance in hyperphagic pseudopregnant rats by chronic prolactin infusion. Endocrinology.

[bib25] Augustine RA, Kokay IC, Andrews ZB, Ladyman SR, Grattan DR (2003). Quantitation of prolactin receptor mRNA in the maternal rat brain during pregnancy and lactation. Journal of Molecular Endocrinology.

[bib26] Augustine RA, Ladyman SR, Grattan DR (2008). From feeding one to feeding many: hormone-induced changes in bodyweight homeostasis during pregnancy. Journal of Physiology.

[bib27] Averill RL, Grattan DR, Norris SK (1991). Posterior pituitary lobectomy chronically attenuates the nocturnal surge of prolactin in early pregnancy. Endocrinology.

[bib291] Bachelot A, Beaufaron J, Servel N, Kedzia C, Monget P, Kelly PA, Gibori G, Binart N (2009). Prolactin independent rescue of mouse corpus luteum life span: identification of prolactin and luteinizing hormone target genes. American Journal of Physiology. Endocrinology and Metabolism.

[bib28] Bakowska JC, Morrell JI (1997). Atlas of the neurons that express mRNA for the long form of the prolactin receptor in the forebrain of the female rat. Journal of Comparative Neurology.

[bib29] Baptista T, de Baptista EA, Lalonde J, Plamondon J, Kin NM, Beaulieu S, Joober R, Richard D (2004). Comparative effects of the antipsychotics sulpiride and risperidone in female rats on energy balance, body composition, fat morphology and macronutrient selection. Progress in Neuro-Psychopharmacology & Biological Psychiatry.

[bib30] Barber MC, Clegg RA, Finley E, Vernon RG, Flint DJ (1992). The role of growth hormone, prolactin and insulin-like growth factors in the regulation of rat mammary gland and adipose tissue metabolism during lactation. Journal of Endocrinology.

[bib31] Barraclough CA, Sawyer CH (1959). Induction of pseudopregnancy in the rat by reserpine and chlorpromazine. Endocrinology.

[bib32] Becquet D, Boyer B, Rasolonjanahary R, Brue T, Guillen S, Moreno M, Franc JL, Francois-Bellan AM (2014). Evidence for an internal and functional circadian clock in rat pituitary cells. Molecular and Cellular Endocrinology.

[bib33] Ben-Jonathan N, Hnasko R (2001). Dopamine as a prolactin (PRL) inhibitor. Endocrine Reviews.

[bib34] Ben-Jonathan N, Oliver C, Weiner HJ, Mical RS, Porter JC (1977). Dopamine in hypophysial portal plasma of the rat during the estrous cycle and throughout pregnancy. Endocrinology.

[bib35] Ben-Jonathan N, Neill MA, Arbogast LA, Peters LL, Hoefer MT (1980). Dopamine in hypophysial portal blood: relationship to circulating prolactin in pregnant and lactating rats. Endocrinology.

[bib36] Ben-Jonathan N, Hugo ER, Brandebourg TD, LaPensee CR (2006). Focus on prolactin as a metabolic hormone. Trends in Endocrinology and Metabolism.

[bib37] Ben-Jonathan N, Lapensee CR, Lapensee EW (2008). What can we learn from rodents about prolactin in humans?. Endocrine Reviews.

[bib38] Berghorn KA, Le WW, Sherman TG, Hoffman GE (2001). Suckling stimulus suppresses messenger RNA for tyrosine hydroxylase in arcuate neurons during lactation. Journal of Comparative Neurology.

[bib39] Bjorklund A, Moore RY, Nobin A, Stenevi U (1973). The organization of tubero-hypophyseal and reticulo-infundibular catecholamine neuron systems in the rat brain. Brain Research.

[bib40] Blum M, McEwen BS, Roberts JL (1987). Transcriptional analysis of tyrosine hydroxylase gene expression in the tuberoinfundibular dopaminergic neurons of the rat arcuate nucleus after estrogen treatment. Journal of Biological Chemistry.

[bib41] Bohnet HG, Dahlen HG, Wuttke W, Schneider HP (1976). Hyperprolactinemic anovulatory syndrome. Journal of Clinical Endocrinology and Metabolism.

[bib42] Bole-Feysot C, Goffin V, Edery M, Binart N, Kelly PA (1998). Prolactin (PRL) and its receptor: actions, signal transduction pathways and phenotypes observed in PRL receptor knockout mice. Endocrine Reviews.

[bib43] Bone AJ, Taylor KW (1976). Metabolic adaptation to pregnancy shown by increased biosynthesis of insulin in islets of Langerhans isolated from pregnant rat. Nature.

[bib44] Borgundvaag B, Kudlow JE, Mueller SG, George SR (1992). Dopamine receptor activation inhibits estrogen-stimulated transforming growth factor-α gene expression and growth in anterior pituitary, but not in uterus. Endocrinology.

[bib45] Bosse R, Fumagalli F, Jaber M, Giros B, Gainetdinov RR, Wetsel WC, Missale C, Caron MG (1997). Anterior pituitary hypoplasia and dwarfism in mice lacking the dopamine transporter. Neuron.

[bib46] Brelje TC, Parsons JA, Sorenson RL (1994). Regulation of islet β-cell proliferation by prolactin in rat islets. Diabetes.

[bib47] Brelje TC, Stout LE, Bhagroo NV, Sorenson RL (2004). Distinctive roles for prolactin and growth hormone in the activation of signal transducer and activator of transcription 5 in pancreatic islets of Langerhans. Endocrinology.

[bib48] Bridges RS, Ronsheim PM (1990). Prolactin (PRL) regulation of maternal behavior in rats: bromocriptine treatment delays and PRL promotes the rapid onset of behavior. Endocrinology.

[bib49] Bridges RS, DiBiase R, Loundes DD, Doherty PC (1985). Prolactin stimulation of maternal behavior in female rats. Science.

[bib50] Bridges RS, Numan M, Ronsheim PM, Mann PE, Lupini CE (1990). Central prolactin infusions stimulate maternal behavior in steroid-treated, nulliparous female rats. PNAS.

[bib51] Bridges RS, Robertson MC, Shiu RPC, Friesen HG, Stuer AM, Mann PE (1996). Endocrine communication between conceptus and mother: placental lactogen stimulation of maternal behavior. Neuroendocrinology.

[bib52] Brown RS, Kokay IC, Herbison AE, Grattan DR (2010). Distribution of prolactin-responsive neurons in the mouse forebrain. Journal of Comparative Neurology.

[bib53] Brown RS, Piet R, Herbison AE, Grattan DR (2012). Differential actions of prolactin on electrical activity and intracellular signal transduction in hypothalamic neurons. Endocrinology.

[bib54] Brown RS, Herbison AE, Grattan DR (2014). Prolactin regulation of kisspeptin neurones in the mouse brain and its role in the lactation-induced suppression of kisspeptin expression. Journal of Neuroendocrinology.

[bib292] Brunton PJ, Russell JA (2008). The expectant brain: adapting for motherhood. Nature Reviews. Neuroscience.

[bib55] Buntin JD, Hnasko RM, Zuzick PH (1999). Role of the ventromedial hypothalamus in prolactin-induced hyperphagia in ring doves. Physiology & Behavior.

[bib56] Carre N, Binart N (2014). Prolactin and adipose tissue. Biochimie.

[bib57] Charoenphandhu N, Wongdee K, Krishnamra N (2010). Is prolactin the cardinal calciotropic maternal hormone?. Trends in Endocrinology and Metabolism.

[bib58] Chen P, Smith MS (2004). Regulation of hypothalamic neuropeptide Y messenger ribonucleic acid expression during lactation: role of prolactin. Endocrinology.

[bib59] Cheung CY (1983). Prolactin suppresses luteinizing hormone secretion and pituitary responsiveness to luteinizing hormone-releasing hormone by a direct action at the anterior pituitary. Endocrinology.

[bib60] Clarkson J, Herbison AE (2009). Oestrogen, kisspeptin, GPR54 and the pre-ovulatory luteinising hormone surge. Journal of Neuroendocrinology.

[bib61] Cohen-Becker IR, Selmanoff M, Wise PM (1986). Hyperprolactinemia alters the frequency and amplitude of pulsatile luteinizing hormone secretion in the ovariectomized rat. Neuroendocrinology.

[bib62] Cowie AT, Tindal JS, Benson GK (1960). Pituitary grafts and milk secretion in hypophysectomized rats. Journal of Endocrinology.

[bib63] Cramer OM, Parker CR, Porter JC (1979). Estrogen inhibition of dopamine release into hypophysial portal blood. Endocrinology.

[bib64] Creemers LB, Zelissen PM, van 't Verlaat JW, Koppeschaar HP (1991). Prolactinoma and body weight: a retrospective study. Acta Endocrinologica.

[bib65] Crowley WR (2015). Neuroendocrine regulation of lactation and milk production. Comprehensive Physiology.

[bib66] Cservenak M, Bodnar I, Usdin TB, Palkovits M, Nagy GM, Dobolyi A (2010). Tuberoinfundibular peptide of 39 residues is activated during lactation and participates in the suckling-induced prolactin release in rat. Endocrinology.

[bib67] Cusan L, Dupont A, Kledzik GS, Labrie F, Coy DH, Schally AV (1977). Potent prolactin and growth hormone releasing activity of more analogues of Met-enkephalin. Nature.

[bib68] De Greef WJ, Neill JD (1979). Dopamine levels in hypophysial stalk plasma of the rat during surges of prolactin secretion induced by cervical stimulation. Endocrinology.

[bib69] De Lean A, Labrie F (1977). Sensitizing effect of treatment with estrogens on TSH response to TRH in male rats. American Journal of Physiology.

[bib70] Delgrange E, Donckier J, Maiter D (1999). Hyperprolactinaemia as a reversible cause of weight gain in male patients?. Clinical Endocrinology.

[bib71] Demarest KT, McKay DW, Riegle GD, Moore KE (1983). Biochemical indices of tuberoinfundibular dopaminergic neuronal activity during lactation: a lack of response to prolactin. Neuroendocrinology.

[bib72] Demarest KT, Riegle GD, Moore KE (1984a). Prolactin-induced activation of tuberoinfundibular dopaminergic neurons: evidence for both a rapid ‘tonic’ and a delayed ‘induction’ component. Neuroendocrinology.

[bib73] Demarest KT, Riegle GD, Moore KE (1984b). Long-term treatment with estradiol induces reversible alterations in tuberoinfundibular dopaminergic neurons: a decreased responsiveness to prolactin. Neuroendocrinology.

[bib74] Demarest KT, Riegle GD, Moore KE (1986). The rapid ‘tonic’ and the delayed ‘induction’ components of the prolactin-induced activation of tuberoinfundibular dopaminergic neurons following the systemic administration of prolactin. Neuroendocrinology.

[bib75] DeMaria JE, Livingstone JD, Freeman ME (1998). Characterization of the dopaminergic input to the pituitary gland throughout the estrous cycle of the rat. Neuroendocrinology.

[bib76] DeMaria JE, Lerant AA, Freeman ME (1999). Prolactin activates all three populations of hypothalamic neuroendocrine dopaminergic neurons in ovariectomized rats. Brain Research.

[bib77] DeMaria JE, Livingstone JD, Freeman ME (2000). Ovarian steroids influence the activity of neuroendocrine dopaminergic neurons. Brain Research.

[bib78] Dempsey EW, Uotila UU (1940). The effect of pituitary stalk section upon reproductive phenomena in the female rat. Endocrinology.

[bib79] Desroziers E, Mikkelsen J, Simonneaux V, Keller M, Tillet Y, Caraty A, Franceschini I (2010). Mapping of kisspeptin fibres in the brain of the pro-oestrous rat. Journal of Neuroendocrinology.

[bib80] Dobolyi A (2011). Novel potential regulators of maternal adaptations during lactation: tuberoinfundibular peptide 39 and amylin. Journal of Neuroendocrinology.

[bib81] Dobolyi A, Dimitrov E, Palkovits M, Usdin TB (2012). The neuroendocrine functions of the parathyroid hormone 2 receptor. Frontiers in Endocrinology.

[bib82] Doknic M, Pekic S, Zarkovic M, Medic-Stojanoska M, Dieguez C, Casanueva F, Popovic V (2002). Dopaminergic tone and obesity: an insight from prolactinomas treated with bromocriptine. European Journal of Endocrinology/European Federation of Endocrine Societies.

[bib83] Eikenburg DC, Ravitz AJ, Gudelsky GA, Moore KE (1977). Effects of estrogen on prolactin and tuberoinfundibular dopaminergic neurons. Journal of Neural Transmission.

[bib84] Elsholtz HP, Lew AM, Albert PR, Sundmark VC (1991). Inhibitory control of prolactin and Pit-1 gene promoters by dopamine. Dual signaling pathways required for D2 receptor-regulated expression of the prolactin gene. Journal of Biological Chemistry.

[bib85] Enjalbert A, Ruberg M, Arancibia S, Priam M, Kordon C (1979). Endogenous opiates block dopamine inhibition of prolactin secretion *in vitro*. Nature.

[bib86] Everett JW (1954). Luteotrophic function of autographs of the rat hypophysis. Endocrinology.

[bib87] Feher P, Olah M, Bodnar I, Hechtl D, Bacskay I, Juhasz B, Nagy GM, Vecsernyes M (2010). Dephosphorylation/inactivation of tyrosine hydroxylase at the median eminence of the hypothalamus is required for suckling-induced prolactin and adrenocorticotrop hormone responses. Brain Research Bulletin.

[bib88] Fink G (1988). Oestrogen and progesterone interactions in the control of gonadotrophin and prolactin secretion. Journal of Steroid Biochemistry.

[bib89] Fliestra RJ, Voogt JL (1997). Lactogenic hormones of the placenta and pituitary inhibit suckling-induced prolactin (PRL) release but not the ante-partum PRL surge. Proceedings of the Society for Experimental Biology and Medicine.

[bib90] Fox SR, Smith MS (1984). The suppression of pulsatile luteinizing hormone secretion during lactation in the rat. Endocrinology.

[bib91] Fox SR, Hoefer MT, Bartke A, Smith MS (1987). Suppression of pulsatile LH secretion, pituitary GnRH receptor content and pituitary responsiveness to GnRH by hyperprolactinemia in the male rat. Neuroendocrinology.

[bib92] Fox SR, Harlan RE, Shivers BD, Pfaff DW (1990). Chemical characterization of neuroendocrine targets for progesterone in the female rat brain and pituitary. Neuroendocrinology.

[bib93] Freeman ME, Kanyicska B, Lerant A, Nagy G (2000). Prolactin: structure, function, and regulation of secretion. Physiological Reviews.

[bib94] Freemark M, Avril I, Fleenor D, Driscoll P, Petro A, Opara E, Kendall W, Oden J, Bridges S, Binart N (2002). Targeted deletion of the PRL receptor: effects on islet development, insulin production, and glucose tolerance. Endocrinology.

[bib95] Furigo IC, Kim KW, Nagaishi VS, Ramos-Lobo AM, de Alencar A, Pedroso JA, Metzger M, Donato J (2014). Prolactin-sensitive neurons express estrogen receptor-α and depend on sex hormones for normal responsiveness to prolactin. Brain Research.

[bib96] Fuxe K (1963). Cellular localisations of monoamines in the median eminence and in the infundibular stem of some mammals. Acta Physiologica Scandinavica.

[bib97] Fuxe K (1964). Cellular localization of monoamines in the median eminence and the infundibular stem of some mammals. Zeitschrift für Zellforschung und Mikroskopische Anatomie. Abteilung Histochemie.

[bib98] Gala RR (1990). The physiology and mechanisms of the stress-induced changes in prolactin secretion in the rat. Life Sciences.

[bib99] Galluzzi F, Salti R, Stagi S, La Cauza F, Chiarelli F (2005). Reversible weight gain and prolactin levels – long-term follow-up in childhood. Journal of Pediatric Endocrinology & Metabolism.

[bib100] Gerardo-Gettens T, Moore BJ, Stern JS, Horwitz BA (1989). Prolactin stimulates food intake in a dose-dependent manner. American Journal of Physiology.

[bib101] Gettler LT, McDade TW, Feranil AB, Kuzawa CW (2012). Prolactin, fatherhood, and reproductive behavior in human males. American Journal of Physical Anthropology.

[bib293] Ghosh R, Sladek CD (1995a). Prolactin modulates oxytocin mRNA during lactation by its action on the hypothalamo-neurohypophyseal axis. Brain Research.

[bib294] Ghosh R, Sladek CD (1995b). Role of prolactin and gonadal steroids in regulation of oxytocin mRNA during lactation. American Journal of Physiology.

[bib102] Gibbs DM, Neill JD (1978). Dopamine levels in hypophysial stalk blood in the rat are sufficient to inhibit prolactin secretion *in vivo*. Endocrinology.

[bib295] Gibori G, Richards JS (1978). Dissociation of two distinct luteotropic effects of prolactin: regulation of luteinizing hormone-receptor content and progesterone secretion during pregnancy. Endocrinology.

[bib103] Goffin V, Binart N, Touraine P, Kelly PA (2002). Prolactin: the new biology of an old hormone. Annual Review of Physiology.

[bib104] Gordon I, Zagoory-Sharon O, Leckman JF, Feldman R (2010). Prolactin, oxytocin, and the development of paternal behavior across the first six months of fatherhood. Hormones and Behavior.

[bib105] Goudreau JL, Lindley SE, Lookingland KJ, Moore KE (1992). Evidence that hypothalamic periventricular dopamine neurons innervate the intermediate lobe of the rat pituitary. Neuroendocrinology.

[bib106] Grattan DR (2001). The actions of prolactin in the brain during pregnancy and lactation. Progress in Brain Research.

[bib107] Grattan DR, Averill RL (1990). Effect of ovarian steroids on a nocturnal surge of prolactin secretion that precedes parturition in the rat. Endocrinology.

[bib108] Grattan DR, Averill RL (1991). Intrahypothalamic pituitary grafts elevate prolactin in the cerebrospinal fluid and attenuate prolactin release following ether stress. Proceedings of the Society for Experimental Biology and Medicine.

[bib109] Grattan DR, Averill RL (1995). Absence of short-loop autoregulation of prolactin during late pregnancy in the rat. Brain Research Bulletin.

[bib110] Grattan DR, Kokay IC (2008). Prolactin: a pleiotropic neuroendocrine hormone. Journal of Neuroendocrinology.

[bib111] Grattan DR & LeTissier P 2015 Hypothalamic control of prolactin secretion, and the multiple reproductive functions of prolactin. In *Knobil and Neill's Physiology of Reproduction*, 4th edn, pp 469–526. Eds TM Plant & AJ Zelesnik. Amsterdam: Elsevier.

[bib112] Grattan DR, Xu J, McLachlan MJ, Kokay IC, Bunn SJ, Hovey RC, Davey HW (2001). Feedback regulation of PRL secretion is mediated by the transcription factor, signal transducer, and activator of transcription 5b. Endocrinology.

[bib113] Grattan DR, Ladyman SR, Augustine RA (2007a). Hormonal induction of leptin resistance during pregnancy. Physiology & Behavior.

[bib114] Grattan DR, Jasoni CL, Liu X, Anderson GM, Herbison AE (2007b). Prolactin regulation of gonadotropin-releasing hormone neurons to suppress luteinizing hormone secretion in mice. Endocrinology.

[bib115] Grattan DR, Steyn FJ, Kokay IC, Anderson GM, Bunn SJ (2008). Pregnancy-induced adaptation in the neuroendocrine control of prolactin secretion. Journal of Neuroendocrinology.

[bib116] Greenman Y, Tordjman K, Stern N (1998). Increased body weight associated with prolactin secreting pituitary adenomas: weight loss with normalization of prolactin levels. Clinical Endocrinology.

[bib117] Gregerson KA (2003). Functional expression of the dopamine-activated K(+) current in lactotrophs during the estrous cycle in female rats: correlation with prolactin secretory responses. Endocrine.

[bib118] Gregerson KA, Neill JD (2006). Prolactin: structure, function, and regulation of secretion. Knobil and Neill's Physiology of Reproduction.

[bib119] Gregerson KA, Golesorkhi N, Chuknyiska R (1994). Stimulation of prolactin release by dopamine withdrawal: role of membrane hyperpolarization. American Journal of Physiology.

[bib120] Gudelsky GA, Porter JC (1979). Release of newly synthesized dopamine into the hypophysial portal vasculature of the rat. Endocrinology.

[bib121] Gunnet JW, Freeman ME (1983). The mating-induced release of prolactin: a unique neuroendocrine response. Endocrine Reviews.

[bib122] Han SK, Gottsch ML, Lee KJ, Popa SM, Smith JT, Jakawich SK, Clifton DK, Steiner RA, Herbison AE (2005). Activation of gonadotropin-releasing hormone neurons by kisspeptin as a neuroendocrine switch for the onset of puberty. Journal of Neuroscience.

[bib123] Harris GW (1948). Neural control of the pituitary gland. Physiological Reviews.

[bib124] Hentges ST, Low MJ (2002). Ovarian dependence for pituitary tumorigenesis in D2 dopamine receptor-deficient mice. Endocrinology.

[bib125] Herbison AE (2008). Estrogen positive feedback to gonadotropin-releasing hormone (GnRH) neurons in the rodent: the case for the rostral periventricular area of the third ventricle (RP3V). Brain Research Reviews.

[bib126] Herrera E (2000). Metabolic adaptations in pregnancy and their implications for the availability of substrates to the fetus. European Journal of Clinical Nutrition.

[bib127] Hnasko R, Khurana S, Shackleford N, Steinmetz R, Low MJ, Ben-Jonathan N (1997). Two distinct pituitary cell lines from mouse intermediate lobe tumors: a cell that produces prolactin-regulating factor and a melanotroph. Endocrinology.

[bib128] Hokfelt T, Fuxe K (1972). Effects of prolactin and ergot alkaloids on the tubero-infundibular dopamine (DA) neurons. Neuroendocrinology.

[bib129] Holzbauer M, Racke K (1985). The dopaminergic innervation of the intermediate lobe and of the neural lobe of the pituitary gland. Medical Biology.

[bib130] Horseman ND (1995). Prolactin, proliferation, and protooncogenes. Endocrinology.

[bib131] Hovey RC, Trott JF, Ginsburg E, Goldhar A, Sasaki MM, Fountain SJ, Sundararajan K, Vonderhaar BK (2001). Transcriptional and spatiotemporal regulation of prolactin receptor mRNA and cooperativity with progesterone receptor function during ductal branch growth in the mammary gland. Developmental Dynamics.

[bib132] Huang C, Snider F, Cross JC (2009). Prolactin receptor is required for normal glucose homeostasis and modulation of β-cell mass during pregnancy. Endocrinology.

[bib133] Huerta-Ocampo I, Christian HC, Thompson NM, El-Kasti MM, Wells T (2005). The Intermediate lactotroph: a morphologically distinct, ghrelin-responsive pituitary cell in the dwarf (dw/dw) rat. Endocrinology.

[bib134] Ishida M, Mitsui T, Yamakawa K, Sugiyama N, Takahashi W, Shimura H, Endo T, Kobayashi T, Arita J (2007). Involvement of cAMP response element-binding protein in the regulation of cell proliferation and the prolactin promoter of lactotrophs in primary culture. American Journal of Physiology. Endocrinology and Metabolism.

[bib135] Johnston CA, Demarest KT, Moore KE (1980). Cycloheximide disrupts the prolactin-mediated stimulation of dopamine synthesis in tuberoinfundibular neurons. Brain Research.

[bib136] Jones EE, Naftolin F (1990). Estrogen effects on the tuberoinfundibular dopaminergic system in the female rat brain. Brain Research.

[bib137] Kamberi IA, Mical RS, Porter JC (1970). Prolactin-inhibiting activity in hypophysial stalk blood and elevation by dopamine. Experientia.

[bib138] Kanematsu S, Sawyer CH (1973). Elevation of plasma prolactin after hypophysial stalk section in the rat. Endocrinology.

[bib139] Kanematsu S, Hilliard J, Sawyer CH (1963). Effect of reserpine on pituitary prolactin content and its hypothalamic site of action in the rabbit. Acta Endocrinologica.

[bib140] Kansra S, Yamagata S, Sneade L, Foster L, Ben-Jonathan N (2005). Differential effects of estrogen receptor antagonists on pituitary lactotroph proliferation and prolactin release. Molecular and Cellular Endocrinology.

[bib141] Kansra S, Chen S, Bangaru ML, Sneade L, Dunckley JA, Ben-Jonathan N (2010). Selective estrogen receptor down-regulator and selective estrogen receptor modulators differentially regulate lactotroph proliferation. PLoS ONE.

[bib142] Karnik SK, Chen H, McLean GW, Heit JJ, Gu X, Zhang AY, Fontaine M, Yen MH, Kim SK (2007). Menin controls growth of pancreatic β-cells in pregnant mice and promotes gestational diabetes mellitus. Science.

[bib143] Kelly MA, Rubinstein M, Asa SL, Zhang G, Saez C, Bunzow JR, Allen RG, Hnasko R, Ben-Jonathan N, Grandy DK (1997). Pituitary lactotroph hyperplasia and chronic hyperprolactinemia in dopamine D2 receptor-deficient mice. Neuron.

[bib144] Kim H, Toyofuku Y, Lynn FC, Chak E, Uchida T, Mizukami H, Fujitani Y, Kawamori R, Miyatsuka T, Kosaka Y (2010). Serotonin regulates pancreatic β cell mass during pregnancy. Nature Medicine.

[bib145] Koike K, Aono T, Miyake A, Tasaka K, Chatani F, Kurachi K (1984). Effect of pituitary transplants on the LH-RH concentrations in the medial basal hypothalamus and hypophyseal portal blood. Brain Research.

[bib146] Koike K, Miyake A, Aono T, Sakumoto T, Ohmichi M, Yamaguchi M, Tanizawa O (1991). Effect of prolactin on the secretion of hypothalamic GnRH and pituitary gonadotropins. Hormone Research.

[bib147] Kokay IC, Grattan DR (2005). Expression of mRNA for prolactin receptor (long form) in dopamine and pro-opiomelanocortin neurones in the arcuate nucleus of non-pregnant and lactating rats. Journal of Neuroendocrinology.

[bib296] Kokay IC, Bull PM, Davis RL, Ludwig M, Grattan DR (2006). Expression of the long form of the prolactin receptor in magnocellular oxytocin neurons is associated with specific prolactin regulation of oxytocin neurons. American Journal of Physiology: Regulatory, Integrative and Comparative Physiology.

[bib148] Kokay IC, Petersen SL, Grattan DR (2011). Identification of prolactin-sensitive GABA and kisspeptin neurons in regions of the rat hypothalamus involved in the control of fertility. Endocrinology.

[bib149] Ladyman SR (2008). Leptin resistance during pregnancy in the rat. Journal of Neuroendocrinology.

[bib150] Ladyman SR, Grattan DR (2004). Region-specific reduction in leptin-induced phosphorylation of signal transducer and activator of transcription-3 (STAT3) in the rat hypothalamus is associated with leptin resistance during pregnancy. Endocrinology.

[bib151] Ladyman SR, Woodside B (2014). Food restriction during lactation suppresses Kiss1 mRNA expression and kisspeptin-stimulated LH release in rats. Reproduction.

[bib152] Ladyman SR, Augustine RA, Grattan DR (2010). Hormone interactions regulating energy balance during pregnancy. Journal of Neuroendocrinology.

[bib153] Ladyman SR, Fieldwick DM, Grattan DR (2012). Suppression of leptin-induced hypothalamic JAK/STAT signalling and feeding response during pregnancy in the mouse. Reproduction.

[bib154] Larsen CM, Grattan DR (2010). Prolactin-induced mitogenesis in the subventricular zone of the maternal brain during early pregnancy is essential for normal *postpartum* behavioral responses in the mother. Endocrinology.

[bib155] Larsen CM, Grattan DR (2012). Prolactin, neurogenesis, and maternal behaviors. Brain, Behavior, and Immunity.

[bib156] Larsen CM, Kokay IC, Grattan DR (2008). Male pheromones initiate prolactin-induced neurogenesis and advance maternal behavior in female mice. Hormones and Behavior.

[bib157] Lecomte P, Lecomte C, Lansac J, Gallier J, Sonier CB, Simonetta C (1997). Pregnancy after intravenous pulsatile gonadotropin-releasing hormone in a hyperprolactinaemic woman resistant to treatment with dopamine agonists. European Journal of Obstetrics, Gynecology, and Reproductive Biology.

[bib158] Lehman MN, Coolen LM, Goodman RL (2010). Minireview: Kisspeptin/neurokinin B/dynorphin (KNDy) cells of the arcuate nucleus: a central node in the control of gonadotropin-releasing hormone secretion. Endocrinology.

[bib159] Lerant A, Freeman ME (1998). Ovarian steroids differentially regulate the expression of prolactin receptors in neuroendocrine dopaminergic neuron populations – a double-label confocal microscopic study. Brain Research.

[bib160] Li C, Chen P, Smith MS (1999). Neuropeptide Y and tuberoinfundibular dopamine activities are altered during lactation: role of prolactin. Endocrinology.

[bib161] Li XF, Kinsey-Jones JS, Cheng Y, Knox AM, Lin Y, Petrou NA, Roseweir A, Lightman SL, Milligan SR, Millar RP (2009). Kisspeptin signalling in the hypothalamic arcuate nucleus regulates GnRH pulse generator frequency in the rat. PLoS ONE.

[bib162] Li Q, Rao A, Pereira A, Clarke IJ, Smith JT (2011). Kisspeptin cells in the ovine arcuate nucleus express prolactin receptor but not melatonin receptor. Journal of Neuroendocrinology.

[bib163] Lieberman ME, Maurer RA, Claude P, Wiklund J, Wertz N, Gorski J (1981). Regulation of pituitary growth and prolactin gene expression by estrogen. Advances in Experimental Medicine and Biology.

[bib164] Lincoln GA, Andersson H, Hazlerigg D (2003). Clock genes and the long-term regulation of prolactin secretion: evidence for a photoperiod/circannual timer in the pars tuberalis. Journal of Neuroendocrinology.

[bib165] Liu B, Arbogast LA (2008). Phosphorylation state of tyrosine hydroxylase in the stalk-median eminence is decreased by progesterone in cycling female rats. Endocrinology.

[bib166] Liu B, Arbogast LA (2010). Progesterone decreases tyrosine hydroxylase phosphorylation state and increases protein phosphatase 2A activity in the stalk-median eminence on proestrous afternoon. Journal of Endocrinology.

[bib167] Liu X, Lee K, Herbison AE (2008). Kisspeptin excites gonadotropin-releasing hormone neurons through a phospholipase C/calcium-dependent pathway regulating multiple ion channels. Endocrinology.

[bib168] Liu X, Brown RS, Herbison AE, Grattan DR (2014). Lactational anovulation in mice results from a selective loss of kisspeptin input to GnRH neurons. Endocrinology.

[bib169] Lledo PM, Legendre P, Israel JM, Vincent JD (1990). Dopamine inhibits two characterized voltage-dependent calcium currents in identified rat lactotroph cells. Endocrinology.

[bib170] Login IS, MacLeod RM (1977). Prolactin in human and rat serum and cerebrospinal fluid. Brain Research.

[bib171] Lonstein JS, Blaustein JD (2004). Immunocytochemical investigation of nuclear progestin receptor expression within dopaminergic neurones of the female rat brain. Journal of Neuroendocrinology.

[bib297] Lonstein JS (2007). Regulation of anxiety during the postpartum period. Frontiers in Neuroendocrinology.

[bib172] Lookingland KJ, Jarry HD, Moore KE (1987). The metabolism of dopamine in the median eminence reflects the activity of tuberoinfundibular neurons. Brain Research.

[bib173] Lucas BK, Ormandy CJ, Binart N, Bridges RS, Kelly PA (1998). Null mutation of the prolactin receptor gene produces a defect in maternal behavior. Endocrinology.

[bib174] Lyons DJ, Horjales-Araujo E, Broberger C (2010). Synchronized network oscillations in rat tuberoinfundibular dopamine neurons: switch to tonic discharge by thyrotropin-releasing hormone. Neuron.

[bib175] Lyons DJ, Hellysaz A, Broberger C (2012). Prolactin regulates tuberoinfundibular dopamine neuron discharge pattern: novel feedback control mechanisms in the lactotrophic axis. Journal of Neuroscience.

[bib176] Ma FY, Grattan DR, Goffin V, Bunn SJ (2005). Prolactin-regulated tyrosine hydroxylase activity and messenger ribonucleic acid expression in mediobasal hypothalamic cultures: the differential role of specific protein kinases. Endocrinology.

[bib177] MacLeod RM, Fontham EH, Lehmeyer JE (1970). Prolactin and growth hormone production as influenced by catecholamines and agents that affect brain catecholamines. Neuroendocrinology.

[bib178] Mak GK, Weiss S (2010). Paternal recognition of adult offspring mediated by newly generated CNS neurons. Nature Neuroscience.

[bib179] Mansour A, Meador-Woodruff JH, Bunzow JR, Civelli O, Akil H, Watson SJ (1990). Localization of dopamine D2 receptor mRNA and D1 and D2 receptor binding in the rat brain and pituitary: an *in situ* hybridization-receptor autoradiographic analysis. Journal of Neuroscience.

[bib180] Martinez de la Escalera G, Weiner RI (1992). Dissociation of dopamine from its receptor as a signal in the pleiotropic hypothalamic regulation of prolactin secretion. Endocrine Reviews.

[bib181] Matsumoto M, Hidaka K, Tada S, Tasaki Y, Yamaguchi T (1995). Full-length cDNA cloning and distribution of human dopamine D4 receptor. Brain Research. Molecular Brain Research.

[bib182] Matsuzaki T, Azuma K, Irahara M, Yasui T, Aono T (1994). Mechanism of anovulation in hyperprolactinemic amenorrhea determined by pulsatile gonadotropin-releasing hormone injection combined with human chorionic gonadotropin. Fertility and Sterility.

[bib183] Maurer RA (1982). Estradiol regulates the transcription of the prolactin gene. Journal of Biological Chemistry.

[bib184] McNeilly AS 1994 Suckling and the control of gonadotropin secretion. In *The Physiology of Reproduction*, 2nd edn, pp 1179–1212. Eds E Knobil & JD Neill. New York: Raven Press.

[bib185] McNeilly AS (2001a). Lactational control of reproduction. Reproduction, Fertility, and Development.

[bib186] McNeilly AS (2001b). Neuroendocrine changes and fertility in breast-feeding women. Progress in Brain Research.

[bib187] Merchenthaler I (1993). Induction of enkephalin in tuberoinfundibular dopaminergic neurons during lactation. Endocrinology.

[bib188] Merchenthaler I (1994). Induction of enkephalin in tuberoinfundibular dopaminergic neurons of pregnant, pseudopregnant, lactating and aged female rats. Neuroendocrinology.

[bib189] Meyre D, Delplanque J, Chevre JC, Lecoeur C, Lobbens S, Gallina S, Durand E, Vatin V, Degraeve F, Proenca C (2009). Genome-wide association study for early-onset and morbid adult obesity identifies three new risk loci in European populations. Nature Genetics.

[bib190] Moldrup A, Petersen ED, Nielsen JH (1993). Effects of sex and pregnancy hormones on growth hormone and prolactin receptor gene expression in insulin-producing cells. Endocrinology.

[bib191] Moore BJ, Gerardo-Gettens T, Horwitz BA, Stern JS (1986). Hyperprolactinemia stimulates food intake in the female rat. Brain Research Bulletin.

[bib192] Morel G, Ouhtit A, Kelly PA (1994). Prolactin receptor immunoreactivity in rat anterior pituitary. Neuroendocrinology.

[bib193] Morel GR, Caron RW, Console GM, Soaje M, Sosa YE, Rodriguez SS, Jahn GA, Goya RG (2009). Estrogen inhibits tuberoinfundibular dopaminergic neurons but does not cause irreversible damage. Brain Research Bulletin.

[bib194] Morrell JI, Rosenthal MF, McCabe JT, Harrington CA, Chikaraishi DM, Pfaff DW (1989). Tyrosine hydroxylase mRNA in the neurons of the tuberoinfundibular region and zona incerta examined after gonadal steroid hormone treatment. Molecular Endocrinology.

[bib195] Moult PJ, Rees LH, Besser GM (1982). Pulsatile gonadotrophin secretion in hyperprolactinaemic amenorrhoea an the response to bromocriptine therapy. Clinical Endocrinology.

[bib196] Murai I, Ben-Jonathan N (1987). Posterior pituitary lobectomy abolishes the suckling-induced rise in prolactin (PRL): evidence for a PRL-releasing factor in the posterior pituitary. Endocrinology.

[bib197] Murai I, Ben-Jonathan N (1990). Acute stimulation of prolactin release by estradiol: mediation by the posterior pituitary. Endocrinology.

[bib198] Murai I, Reichlin S, Ben-Jonathan N (1989). The peak phase of the proestrous prolactin surge is blocked by either pituitary lobectomy or antisera to vasoactive intestinal peptide. Endocrinology.

[bib199] Naef L, Woodside B (2007). Prolactin/leptin interactions in the control of food intake in rats. Endocrinology.

[bib200] Nahi F, Arbogast LA (2003). Prolactin modulates hypothalamic preproenkephalin, but not proopiomelanocortin, gene expression during lactation. Endocrine.

[bib201] Navarro VM, Castellano JM, McConkey SM, Pineda R, Ruiz-Pino F, Pinilla L, Clifton DK, Tena-Sempere M, Steiner RA (2011). Interactions between kisspeptin and neurokinin B in the control of GnRH secretion in the female rat. American Journal of Physiology. Endocrinology and Metabolism.

[bib202] Newbern D, Freemark M (2011). Placental hormones and the control of maternal metabolism and fetal growth. Current Opinion in Endocrinology, Diabetes, and Obesity.

[bib203] Nicholson G, Greeley GH, Humm J, Youngblood WW, Kizer JS (1980). Prolactin in cerebrospinal fluid: a probable site of prolactin autoregulation. Brain Research.

[bib204] Nikitovitch-Winer M, Everett JW (1958). Functional restitution of pituitary grafts re-transplanted from kidney to median eminence. Endocrinology.

[bib205] Nilsson L, Olsson AH, Isomaa B, Groop L, Billig H, Ling C (2011). A common variant near the PRL gene is associated with increased adiposity in males. Molecular Genetics and Metabolism.

[bib206] Noel MB, Woodside B (1993). Effects of systemic and central prolactin injections on food intake, weight gain, and estrous cyclicity in female rats. Physiology & Behavior.

[bib207] Nolan LA, Levy A (2009). The trophic effects of oestrogen on male rat anterior pituitary lactotrophs. Journal of Neuroendocrinology.

[bib208] Oakley AE, Clifton DK, Steiner RA (2009). Kisspeptin signaling in the brain. Endocrine Reviews.

[bib209] Ormandy CJ, Binart N, Kelly PA (1997). Mammary gland development in prolactin receptor knockout mice. Journal of Mammary Gland Biology and Neoplasia.

[bib210] Park SK, Selmanoff M (1991). Dose-dependent suppression of postcastration luteinizing hormone secretion exerted by exogenous prolactin administration in male rats: a model for studying hyperprolactinemic hypogonadism. Neuroendocrinology.

[bib211] Park SK, Keenan MW, Selmanoff M (1993). Graded hyperprolactinemia first suppresses LH pulse frequency and then pulse amplitude in castrated male rats. Neuroendocrinology.

[bib298] Parker SL, Armstrong WE, Sladek CD, Grosvenor CE, Crowley WR (1991). Prolactin stimulates the release of oxytocin in lactating rats: evidence for a physiological role via an action at the neural lobe. Neuroendocrinology.

[bib212] Parsons JA, Brelje TC, Sorenson RL (1992). Adaptation of islets of Langerhans to pregnancy: increased islet cell proliferation and insulin secretion correlates with the onset of placental lactogen secretion. Endocrinology.

[bib213] Pasqualini C, Guibert B, Leviel V (1993). Short-term inhibitory effect of estradiol on tyrosine hydroxylase activity in tuberoinfundibular dopaminergic neurons *in vitro*. Journal of Neurochemistry.

[bib214] Pasteels JL (1963). Morphological and experimental research on prolactin secretion. Archives de Biologie.

[bib215] Patel SS, Bamigboye V (2007). Hyperprolactinaemia. Journal of Obstetrics and Gynaecology.

[bib216] Peters LL, Hoefer MT, Ben-Jonathan N (1981). The posterior pituitary: regulation of anterior pituitary prolactin secretion. Science.

[bib217] Petryk A, Fleenor D, Driscoll P, Freemark M (2000). Prolactin induction of insulin gene expression: the roles of glucose and glucose transporter-2. Journal of Endocrinology.

[bib218] Pi XJ, Grattan DR (1998a). Differential expression of the two forms of prolactin receptor mRNA within microdissected hypothalamic nuclei of the rat. Brain Research. Molecular Brain Research.

[bib219] Pi XJ, Grattan DR (1998b). Distribution of prolactin receptor immunoreactivity in the brain of estrogen-treated, ovariectomized rats. Journal of Comparative Neurology.

[bib220] Pinilla L, Aguilar E, Dieguez C, Millar RP, Tena-Sempere M (2012). Kisspeptins and reproduction: physiological roles and regulatory mechanisms. Physiological Reviews.

[bib221] van den Pol AN, Herbst RS, Powell JF (1984). Tyrosine hydroxylase-immunoreactive neurons of the hypothalamus: a light and electron microscopic study. Neuroscience.

[bib222] Polson DW, Sagle M, Mason HD, Adams J, Jacobs HS, Franks S (1986). Ovulation and normal luteal function during LHRH treatment of women with hyperprolactinaemic amenorrhoea. Clinical Endocrinology.

[bib223] Ramos-Roman MA (2011). Prolactin and lactation as modifiers of diabetes risk in gestational diabetes. Hormone and Metabolic Research.

[bib224] Raymond V, Beaulieu M, Labrie F, Boissier J (1978). Potent antidopaminergic activity of estradiol at the pituitary level on prolactin release. Science.

[bib225] Rieck S, Kaestner KH (2010). Expansion of β-cell mass in response to pregnancy. Trends in Endocrinology and Metabolism.

[bib226] Romano N, Yip SH, Hodson DJ, Guillou A, Parnaudeau S, Kirk S, Tronche F, Bonnefont X, Le Tissier P, Bunn SJ (2013). Plasticity of hypothalamic dopamine neurons during lactation results in dissociation of electrical activity and release. Journal of Neuroscience.

[bib227] Rosenblatt JS (1967). Nonhormonal basis of maternal behavior in the rat. Science.

[bib228] Roseweir AK, Kauffman AS, Smith JT, Guerriero KA, Morgan K, Pielecka-Fortuna J, Pineda R, Gottsch ML, Tena-Sempere M, Moenter SM (2009). Discovery of potent kisspeptin antagonists delineate physiological mechanisms of gonadotropin regulation. Journal of Neuroscience.

[bib229] de Roux N, Genin E, Carel JC, Matsuda F, Chaussain JL, Milgrom E (2003). Hypogonadotropic hypogonadism due to loss of function of the KiSS1-derived peptide receptor GPR54. PNAS.

[bib230] Saiardi A, Bozzi Y, Baik JH, Borrelli E (1997). Antiproliferative role of dopamine: loss of D2 receptors causes hormonal dysfunction and pituitary hyperplasia. Neuron.

[bib231] Sakaguchi K, Tanaka M, Ohkubo T, Dohura K, Fujikawa T, Sudo S, Nakashima K (1996). Induction of brain prolactin receptor long-form mRNA expression and maternal behavior in pup-contacted male rats: promotion by prolactin administration and suppression by female contact. Neuroendocrinology.

[bib232] Samson WK, Martin L, Mogg RJ, Fulton RJ (1990). A nonoxytocinergic prolactin releasing factor and a nondopaminergic prolactin inhibiting factor in bovine neurointermediate lobe extracts: *in vitro* and *in vivo* studies. Endocrinology.

[bib299] Sapsford TJ, Kokay IC, Ostberg L, Bridges RS, Grattan DR (2011). Differential sensitivity of specific neuronal populations of the rat hypothalamus to prolactin action. Journal of Comparative Neurology.

[bib233] Sapsford TJ, Kokay IC, Ostberg L, Bridges RS, Grattan DR (2012). Differential sensitivity of specific neuronal populations of the rat hypothalamus to prolactin action. Journal of Comparative Neurology.

[bib234] Sar M (1984). Estradiol is concentrated in tyrosine hydroxylase-containing neurons of the hypothalamus. Science.

[bib235] Sar M (1988). Distribution of progestin-concentrating cells in rat brain: colocalization of [3H]ORG.2058, a synthetic progestin, and antibodies to tyrosine hydroxylase in hypothalamus by combined autoradiography and immunocytochemistry. Endocrinology.

[bib236] Sarkar DK, Frautschy SA, Mitsugi N (1992). Pituitary portal plasma levels of oxytocin during the estrous cycle, lactation, and hyperprolactinemia. Annals of the New York Academy of Sciences.

[bib237] Sauvé D, Woodside B (1996). The effect of central administration of prolactin on food intake in virgin female rats is dose-dependent, occurs in the absence of ovarian hormones and the latency to onset varies with feeding regimen. Brain Research.

[bib238] Sauvé D, Woodside B (2000). Neuroanatomical specificity of prolactin-induced hyperphagia in virgin female rats. Brain Research.

[bib239] Schaeffer M, Langlet F, Lafont C, Molino F, Hodson DJ, Roux T, Lamarque L, Verdie P, Bourrier E, Dehouck B (2013). Rapid sensing of circulating ghrelin by hypothalamic appetite-modifying neurons. PNAS.

[bib240] Schradin C (2007). Comments to K.E. Wynne-Edwards and M.E. Timonin 2007. Paternal care in rodents: weakening support of hormonal regulation of the transition to behavioral fatherhood in rodent animal models of biparental care, Horm & Behav 52: 114-121. Hormones and Behavior.

[bib241] Schradin C, Anzenberger G (1999). Prolactin, the hormone of paternity. News in Physiological Sciences.

[bib242] Schraenen A, Lemaire K, de Faudeur G, Hendrickx N, Granvik M, Van Lommel L, Mallet J, Vodjdani G, Gilon P, Binart N (2010). Placental lactogens induce serotonin biosynthesis in a subset of mouse β cells during pregnancy. Diabetologia.

[bib243] Scully KM, Gleiberman AS, Lindzey J, Lubahn DB, Korach KS, Rosenfeld MG (1997). Role of estrogen receptor-α in the anterior pituitary gland. Molecular Endocrinology.

[bib244] Selmanoff M (1985). Rapid effects of hyperprolactinemia on basal prolactin secretion and dopamine turnover in the medial and lateral median eminence. Endocrinology.

[bib245] Selmanoff M, Gregerson KA (1985). Suckling decreases dopamine turnover in both medial and lateral aspects of the median eminence in the rat. Neuroscience Letters.

[bib246] Selmanoff M, Wise PM (1981). Decreased dopamine turnover in the median eminence in response to suckling in the lactating rat. Brain Research.

[bib247] Selmanoff M, Shu C, Petersen SL, Barraclough CA, Zoeller RT (1991). Single cell levels of hypothalamic messenger ribonucleic acid encoding luteinizing hormone-releasing hormone in intact, castrated, and hyperprolactinemic male rats. Endocrinology.

[bib248] Seminara SB, Messager S, Chatzidaki EE, Thresher RR, Acierno JS, Shagoury JK, Bo-Abbas Y, Kuohung W, Schwinof KM, Hendrick AG (2003). The GPR54 gene as a regulator of puberty. New England Journal of Medicine.

[bib300] Shanks N, Windle RJ, Perks P, Wood S, Ingram CD, Lightman SL (1999). The hypothalamic–pituitary–adrenal axis response to endotoxin is attenuated during lactation. Journal of Neuroendocrinology.

[bib249] Shingo T, Gregg C, Enwere E, Fujikawa H, Hassam R, Geary C, Cross JC, Weiss S (2003). Pregnancy-stimulated neurogenesis in the adult female forebrain mediated by prolactin. Science.

[bib250] Shirley B (1984). The food intake of rats during pregnancy and lactation. Laboratory Animal Science.

[bib251] Short RV (1976). Lactation – the central control of reproduction. Ciba Foundation Symposium.

[bib252] Sinha YN, Sorenson RL (1993). Differential effects of glycosylated and nonglycosylated prolactin on islet cell division and insulin secretion. Proceedings of the Society for Experimental Biology and Medicine.

[bib301] Slattery DA, Neumann ID (2008). No stress please! Mechanisms of stress hyporesponsiveness of the maternal brain. Journal of Physiology.

[bib253] Smith MS (1978). A comparison of pituitary responsiveness to luteinizing hormone-releasing hormone during lactation and the estrous cycle of the rat. Endocrinology.

[bib254] Smith MS (1982). Effect of pulsatile gonadotropin-releasing hormone on the release of luteinizing hormone and follicle-stimulating hormone *in vitro* by anterior pituitaries from lactating and cycling rats. Endocrinology.

[bib255] Smith MS, True C, Grove KL (2010). The neuroendocrine basis of lactation-induced suppression of GnRH: role of kisspeptin and leptin. Brain Research.

[bib256] Sonigo C, Bouilly J, Carre N, Tolle V, Caraty A, Tello J, Simony-Conesa FJ, Millar R, Young J, Binart N (2012). Hyperprolactinemia-induced ovarian acyclicity is reversed by kisspeptin administration. Journal of Clinical Investigation.

[bib257] Sorenson RL, Parsons JA (1985). Insulin secretion in mammosomatotropic tumor-bearing and pregnant rats. A role for lactogens. Diabetes.

[bib258] Sorenson RL, Stout LE (1995). Prolactin receptors and JAK2 in islets of Langerhans: an immunohistochemical analysis. Endocrinology.

[bib259] Steyn FJ, Anderson GM, Grattan DR (2007). Expression of ovarian steroid hormone receptors in tuberoinfundibular dopaminergic neurones during pregnancy and lactation. Journal of Neuroendocrinology.

[bib260] Steyn FJ, Anderson GM, Grattan DR (2008). Hormonal regulation of suppressors of cytokine signaling (SOCS) messenger ribonucleic acid in the arcuate nucleus during late pregnancy. Endocrinology.

[bib261] Szabo FK, Le WW, Snyder NS, Hoffman GE (2011). Comparison of the temporal programs regulating tyrosine hydroxylase and enkephalin expressions in TIDA neurons of lactating rats following pup removal and then pup return. Journal of Molecular Neuroscience.

[bib262] Takahashi S, Okazaki K, Kawashima S (1984). Mitotic activity of prolactin cells in the pituitary glands of male and female rats of different ages. Cell and Tissue Research.

[bib263] Talwalker PK, Ratner A, Meites J (1963). *In vitro* inhibition of pituitary prolactin synthesis and release by hypothalamic extract. American Journal of Physiology.

[bib302] Torner L, Neumann ID (2002). The brain prolactin system: involvement in stress response adaptations in lactation. Stress.

[bib303] Torner L, Toschi N, Pohlinger A, Landgraf R, Neumann ID (2001). Anxiolytic and anti-stress effects of brain prolactin: improved efficacy of antisense targeting of the prolactin receptor by molecular modeling. Journal of Neuroscience.

[bib264] Tortonese DJ, Brooks J, Ingleton PM, McNeilly AS (1998). Detection of prolactin receptor gene expression in the sheep pituitary gland and visualization of the specific translation of the signal in gonadotrophs. Endocrinology.

[bib265] Trott JF, Schennink A, Petrie WK, Manjarin R, VanKlompenberg MK, Hovey RC (2012). Triennial Lactation Symposium: Prolactin: the multifaceted potentiator of mammary growth and function. Journal of Animal Science.

[bib266] True C, Kirigiti M, Ciofi P, Grove KL, Smith MS (2011). Characterisation of arcuate nucleus kisspeptin/neurokinin B neuronal projections and regulation during lactation in the rat. Journal of Neuroendocrinology.

[bib267] Tsukamura H, Maeda K (2001). Non-metabolic and metabolic factors causing lactational anestrus: rat models uncovering the neuroendocrine mechanism underlying the suckling-induced changes in the mother. Progress in Brain Research.

[bib268] Valeggia C, Ellison PT (2009). Interactions between metabolic and reproductive functions in the resumption of *postpartum* fecundity. American Journal of Human Biology.

[bib269] Valerio A, Alberici A, Tinti C, Spano P, Memo M (1994). Antisense strategy unravels a dopamine receptor distinct from the D2 subtype, uncoupled with adenylyl cyclase, inhibiting prolactin release from rat pituitary cells. Journal of Neurochemistry.

[bib270] Walsh RJ, Posner BI, Kopriwa BM, Brawer JR (1978). Prolactin binding sites in the rat brain. Science.

[bib271] Walsh RJ, Slaby FJ, Posner BI (1987). A receptor-mediated mechanism for the transport of prolactin from blood to cerebrospinal fluid. Endocrinology.

[bib272] Walsh RJ, Mangurian LP, Posner BI (1990). Prolactin receptors in the primate choroid plexus. Journal of Anatomy.

[bib273] Wang HJ, Hoffman GE, Smith MS (1993). Suppressed tyrosine hydroxylase gene expression in the tuberoinfundibular dopaminergic system during lactation. Endocrinology.

[bib274] Weber RF, de Greef WJ, de Koning J, Vreeburg JT (1983). LH-RH and dopamine levels in hypophysial stalk plasma and their relationship to plasma gonadotrophins and prolactin levels in male rats bearing a prolactin- and adrenocorticotrophin-secreting pituitary tumor. Neuroendocrinology.

[bib275] Weinhaus AJ, Stout LE, Bhagroo NV, Brelje TC, Sorenson RL (2007). Regulation of glucokinase in pancreatic islets by prolactin: a mechanism for increasing glucose-stimulated insulin secretion during pregnancy. Journal of Endocrinology.

[bib276] West B, Dannies PS (1980). Effects of estradiol on prolactin production and dihydroergocryptine-induced inhibition of prolactin production in primary cultures of rat pituitary cells. Endocrinology.

[bib277] Wongdee K, Tulalamba W, Thongbunchoo J, Krishnamra N, Charoenphandhu N (2011). Prolactin alters the mRNA expression of osteoblast-derived osteoclastogenic factors in osteoblast-like UMR106 cells. Molecular and Cellular Biochemistry.

[bib278] Woodside B (2007). Prolactin and the hyperphagia of lactation. Physiology & Behavior.

[bib279] Woodside B, Budin R, Wellman MK, Abizaid A (2012). Many mouths to feed: the control of food intake during lactation. Frontiers in Neuroendocrinology.

[bib280] Wynne-Edwards KE, Timonin ME (2007). Paternal care in rodents: weakening support for hormonal regulation of the transition to behavioral fatherhood in rodent animal models of biparental care. Hormones and Behavior.

[bib281] Yamada S, Uenoyama Y, Kinoshita M, Iwata K, Takase K, Matsui H, Adachi S, Inoue K, Maeda KI, Tsukamura H (2007). Inhibition of metastin (kisspeptin-54)-GPR54 signaling in the arcuate nucleus-median eminence region during lactation in rats. Endocrinology.

[bib282] Yamada S, Uenoyama Y, Deura C, Minabe S, Naniwa Y, Iwata K, Kawata M, Maeda KI, Tsukamura H (2012). Oestrogen-dependent suppression of pulsatile luteinising hormone secretion and kiss1 mRNA expression in the arcuate nucleus during late lactation in rats. Journal of Neuroendocrinology.

[bib283] Yen SH, Pan JT (1998). Progesterone advances the diurnal rhythm of tuberoinfundibular dopaminergic neuronal activity and the prolactin surge in ovariectomized, estrogen-primed rats and in intact proestrous rats. Endocrinology.

[bib284] Zhang H, Zhang J, Pope CF, Crawford LA, Vasavada RC, Jagasia SM, Gannon M (2010). Gestational diabetes mellitus resulting from impaired β-cell compensation in the absence of FoxM1, a novel downstream effector of placental lactogen. Diabetes.

